# Study on the Effect of PDA-PLGA Scaffold Loaded With Islet Cells for Skeletal Muscle Transplantation in the Treatment of Diabetes

**DOI:** 10.3389/fbioe.2022.927348

**Published:** 2022-06-30

**Authors:** Meishuang Zhang, Hongwei Du, Yueqi Guan, Jingyue Liu, Sushan Wang, Haoran Li, Wenyou Zhang, Hao Han, Ming Zhang, Li Chen

**Affiliations:** ^1^ Department of Pharmacology, College of Basic Medical Sciences, School of Nursing, Jilin University, Changchun, China; ^2^ Research Institution of Paediatrics, Department of Pediatric Endocrinology, The First Clinical Hospital Affiliated to Jilin University, Changchun, China

**Keywords:** electrospinning, PDA-PLGA scaffold, biocompatibility, type Ι diabetes, transplantation in skeletal muscle

## Abstract

At present, islet cells transplantation was limited by the way in which islet cells are implanted into the body, their ability to adapt to the microenvironment and the maintenance time for relieving diabetic symptoms. In order to solve this problem, we made PDA-PLGA scaffold loaded with islet cells and used it for skeletal muscle transplantation to investigate its therapeutic effect in the treatment of diabetes. The PLGA scaffold was prepared by the electrospinning method, and modified by polydopamine coating. A rat diabetic model was established to evaluate the efficacy of PDA-PLGA scaffold loaded with RINm5f islet cells through skeletal muscle transplantation. The results showed that the PDA-PLGA scaffold has good biosafety performance. At the same time, transplantation of the stent to the skeletal muscle site had little effect on the serum biochemical indicators of rats, which was conducive to angiogenesis. The PDA-PLGA scaffold had no effect on the secretory function of pancreatic islet cells. The PDA-PLGA scaffold carrying RINm5f cells was transplanted into the skeletal muscle of type I diabetic rats. 1 week after the transplantation of the PDA-PLGA cell scaffold complex, the blood glucose of the treatment group was significantly lower than that of the model group (*p* < 0.001) and lasted for approximately 3 weeks, which further indicated the skeletal muscle transplantation site was a new choice for islet cell transplantation in the future.

## Introduction

Diabetes is a complex disorder of glucose and lipid metabolism, with an increasing incidence (Roden, 2016). Islet β-cell failure is the decisive factor in type Ι diabetes and the late stage of type II diabetes. At present, drugs for the treatment of diabetes do not induce islet β-cell regeneration and cannot fundamentally cure the disease. With the development of tissue engineering technology, a series of various types of scaffolds apply to improvement and treatment of diseases (Shen et al., 2022), islet cell transplantation provides a new method for the treatment of diabetes (Rengifo et al., 2014; Frei et al., 2018; Perez-Basterrechea et al., 2018). However, its clinical application is limited by the way in which islet cells are implanted into the body, their ability to adapt to the microenvironment and the maintenance time for relieving diabetic symptoms.

Poly (lactic acid-glycolic acid) copolymer (PLGA), as the most widely used polymer material in the biomedical field, has a suitable degradation rate and is nontoxic and harmless, and functional modification of the surface can improve the surface hydrophilicity and histocompatibility (Middleton and Tipton, 2000; Qian et al., 2014). Polydopamine (PDA) is widely used in surface functional modification. It can significantly improve the hydrophilicity and mechanical properties of materials, is nontoxic and biodegradable and has excellent applicability for cell adhesion and proliferation (Lee et al., 2007; Hong et al., 2011; Zhao et al., 2017). There are many studies showing the enhanced biocompatibility effect of polydopamine coat on scaffolds (also on PLGA in bone tissue engineering scaffolds) (Zhao et al., 2017; Yang et al., 2020). Materials can also be designed to prevent adhesive interactions of leukocytes and endothelial cells that ameliorate the microenvironment (Tu et al., 2022).

The selection of the transplantation site is the key to the success of islet cell transplantation. At present, the common transplantation sites mainly include subcutaneous sites, the portal vein and skeletal muscle (Rajab, 2010; Salazar-Bañuelos et al., 2010; Perez et al., 2011). Subcutaneous transplantation cannot meet the requirements of islet cells for the microenvironment because it is insensitive to changes in glucose levels (Cantarelli et al., 2009; Vlahos et al., 2017). Portal vein islet transplantation is prone to immediate blood-mediated inflammation (Bennet et al., 2000). Skeletal muscle, as the key target organ of glucose utilization, can stimulate islet cells in the transplantation site to release insulin over time according to the glucose content. Therefore, skeletal muscle is the preferred site for islet cell transplantation (Christoffersson et al., 2011). However, there is no report on the related research of PDA-PLGA scaffold loaded with islet cells in skeletal muscle transplantation to treat diabetes.

In this study, PLGA scaffolds were prepared by electrospinning and modified by polydopamine coating on the surfaces. The morphology and properties of the scaffolds were identified, and the safety of the scaffolds was evaluated; then, they were cocultured with islet cells to detect the effect of the scaffolds on the morphology and function of islet cells. Furthermore, PDA-PLGA scaffold loaded with islet cells were transplanted into the skeletal muscle of type Ι diabetic rats to explore the therapeutic effect of islet cells and the PDA-PLGA scaffold complex on diabetes mellitus.

## Materials and Methods

### Synthesis of the PLGA and PDA-PLGA Scaffolds

First, the solid polymer PLGA particles, which are the main bodies used for electrospinning, were dissolved in a mixed solution of CHCL_3_ and C_3_H_6_O (v/v = 3:1) and stirred at 25°C for 1 h until completely dissolved, and an electrospinning solution with a mass fraction of 10%–15% was prepared. The electrospinning solution was added to a 5 ml syringe. The front end is connected with the electrospinning machine through a 17G flat head metal spinneret, and the syringe is fixed on the injection pump. The inner diameter of the metal spinneret is 1.12 mm. The distance between the metal receiving plate and the spinneret is adjusted to determine the receiving distance of 15 cm. During the spinning process, the high electrostatic voltage between the metal spinneret and the receiving plate was 15 kV, the flow rate of the electrospinning solution was controlled by an injection pump at 1.5 ml/h for 3 h, and the prepared material was lowered and dried overnight in a ventilator to obtain the PLGA scaffolds.

To generate a polydopamine-coated PDA-PLGA scaffold, a 10 mM Tris-HCl buffer solution of with a pH of 8.5 was prepared, and 20 mg dopamine hydrochloride was dissolved in 10 ml Tris-HCl buffer solution to prepare a weakly alkaline 2 mg/ml dopamine solution. The PLGA scaffold was immersed in a weak alkaline solution of dopamine and kept in aerobic conditions. The dopamine self-polymerized on the surface of the material and was fully shaken to synthesize the polydopamine (PDA) coating. During the self-polymerization of PDA, it was observed that the solution colour changed from light brown to black. After 24 h, the scaffold was removed and washed with distilled water 3 times to remove the unadhered polydopamine molecules. The PDA-PLGA scaffold was obtained by drying the material in a ventilator overnight.

### Structure and Surface Characterization

The surface topography of the PLGA and PDA-PLGA scaffolds was characterized using a field emission scanning electron microscope (FE-SEM; JSM-7401F, JEOL Ltd. Japan), and the surface functional groups were measured by Fourier transform infrared (FTIR) spectroscopy. The contact angles of the samples were investigated using a contact angle metre (SL200B, Solon Technology Science, Shanghai, China) as described previously (Vlahos et al., 2017).

### Mechanical Properties

Rectangular (10 mm × 30 mm) samples with an average thickness of 0.10 mm were tested for tensile strength and the elastic modulus using an electronic universal testing machine (INSTRON 5948, United States). During measurement, the square clamping position of 1 cm × 1 cm was set aside at each end, and sand paper was used to clamp both ends of the samples to prevent slipping during the testing. The crosshead speed was set at 1 mm/min. The yield point value of the stress-strain curve was determined as the tensile strength, and the elasticity modulus was calculated by the slope of the linear portion of the curve.

### Detection of Degradation Behaviour *in vitro*

The prepared PLGA and PDA-PLGA scaffolds were cut into 2.5 × 2.5 cm^2^ squares, and the thickness was measured with a Vernier calliper at four random points on each membrane; the average value was considered the thickness (≈0.1 mm), and the fibre scaffold was placed into a 50 ml centrifuge tube and immersed in 40 ml aseptic PBS buffer solution. It was incubated at 37°C, and the lactic acid level was detected in the solution every week.

### Cell Culture and Proliferation

HUVECs and RAW264.7 cells were cultured in RPMI 1640 medium (Gibco, United States) and 10% foetal bovine serum (Gibco, United States) in an atmosphere of 5% CO_2_ at 37°C. ADSCs, C2C12 and RINm5f cells were cultured in Dulbecco’s modified Eagle’s medium (DMEM) and 10% foetal bovine serum (Gibco, United States) in an atmosphere of 5% CO_2_ at 37°C. MTT (Sigma, United States) was used for the cell proliferation study.

### Cell Morphology and Cell Adhesion

Cells (2×10^4^) were seeded onto 14 mm scaffolds (average thickness of 0.10 mm) and cultured in 24-well plates in an atmosphere of 5% CO_2_ at 37°C for 24 h. After 24 h, the scaffolds carrying the cells were washed with PBS once and fixed with 4% paraformaldehyde at room temperature for 30 min. The sample was washed with PBS 3 times after fixation. Then, the cells were permeated with 0.1% Triton X-100 for 3 min and washed with PBS 3 times after osmosis. Then, 200 μL of phalloidin-iFluor488 reagent (diluted 1:1,000) was added to each well, incubated at room temperature without light for 40 min, and washed with PBS 3 times to remove excess dye. The adhesion morphology of cells on the scaffold was observed by positive fluorescence microscopy (CarlZeiss, Axio Imager Z2). Cells cultured without scaffolds were defined as the control group (Con), cells cocultured with PLGA scaffolds were defined as the PLGA group (PLGA), cells cocultured with PDA-PLGA scaffolds were defined as the PDA-PLGA group (PDA-PLGA).

### Determination of Glucose Consumption and Glucose Uptake in C2C12 Cells

Differentiated C2C12 cells were incubated with conditioned medium obtained from the RINm5f cells seeded onto the PDA-PLGA scaffolds for 24 h. Cells were then cultured in FBS-free DMEM (15 mM D-glucose) supplemented with 0.2% BSA for 48 h. The media glucose concentration was determined as described above. Glucose consumption was calculated based on the difference between the initial glucose concentration and the residual glucose concentration of the culture medium. For glucose uptake determination, cells were washed three times with KRB and incubated with conditioned medium or normal medium for 10 min, and 100 μM 2-NBDG (Invitrogen, United States) was added to the medium for 30 min. The medium was removed, and the cells were then washed twice with ice-cold PBS. The fluorescence intensity in each well was then measured at 485 nm/535 nm (excitation wavelength/emission wavelength) using an Epoch fluorescence microplate reader (Biotek, United States). Cells cultured without conditioned medium were defined as the control group (Con), cells cocultured with conditioned medium were defined as the RINm5f group (RINm5f).

### Determination of Glucose-Stimulated Insulin Secretion

When the RINm5f cells were seeded onto the 14 mm scaffolds (average thickness of 0.10 mm) and inoculated in a 24-well plate until the cell fusion degree reached approximately 80%, the cells were incubated with glucose-free KRB buffer for 90 min, and then the cells were treated according to their respective groupings. KRB buffers containing different concentrations of glucose were added to stimulate the cells for 2 h. After stimulation, the supernatant was collected immediately to terminate the incubation, and the detection of insulin in the supernatant was performed with an ELISA kit.

### Experimental Animals

One hundred and two male 5-6-week-old Wistar rats were purchased from Beijing HFK Bioscience Co., Ltd. [SCXK (Jing) 2019–0008]. They were free fed individually in a room (15–25°C) with a 12 h day-night cycle. After adaptive feeding for 1 week, seventy-two male rats were used to evaluate the safety of the PDA-PLGA scaffold *in vivo*. They were randomly divided into three groups (24 rats in each group were divided into four groups according to different time points, with six rats in each group). Animals without PDA-PLGA scaffold transplantation were defined as control group. PDA-PLGA scaffold was transplanted intraperitoneally (Ip), into skeletal muscle (Mus) and subcutaneously (Sub). Some of the rats were sacrificed on the 3rd, 7th, 14th, and 28th days after transplantation, and related tests were performed.

The remaining thirty rats were used to establish type Ι diabetes models to further detect the therapeutic effect of skeletal muscle transplantation of the PDA-PLGA scaffold loaded with islet cells. After 1 week of acclimation with free access to regular rodent chow and water, the rats were randomly divided into three groups: Group 1 (*n* = 6, normal control [Con]), Group 2 (*n* = 6, diabetic model [DM]) and Group 3 (*n* = 18, treatment group [PDA-PLGA]). After fasting for more than 12 h and injection of high-dose STZ (65 mg/kg) in Group 2 and Group 3, Group 1 was injected with the citrate buffer vehicle. One week after STZ injection, the rats in Group 2 and Group 3 had an FBG level ≥11.1 mmol/L. After the successful establishment of the model, skeletal muscle transplantation of the PDA-PLGA scaffold loaded with islet cells was performed in Group 3, and the fasting blood glucose was detected weekly. After 4 weeks, blood samples obtained from the abdominal aorta were collected in EDTA tubes and placed on ice. The main organs and pancreatic tissues were preserved and stored at −80°C. In this manuscript, the ARRIVE guidelines have been followed. All animal experimental procedures were approved by the Ethics Committee for the Use of Experimental Animals of Jilin University [SYXK (Ji) 2018–0001].

### Biochemical Indicator Detection

At the end of the experimental period, AST, ALT, TP, ALB, ALP, BUN, CR, UA, TNF-α, and IL-4 in the blood and lactic acid levels in the blood and tissue from transplantation sites in rats were measured by ELISA.

### Histopathology

Tissue from the heart, liver, spleen, lung, kidney, and the transplant site were obtained for histopathological examination. Four percent formaldehyde fixation, paraffin embedding, HE staining and Masson trichrome staining were performed.

### Immunofluorescence

Paraffin tissue sections were prepared and incubated with a primary antibody mixture of insulin (1:150, Abcam) at 4°C overnight. Then, the slides were protected from light, and a secondary antibody mixture was used that included anti-mouse IgG FITC (1:100, Sungene) for 1 h at room temperature. Counterstaining was followed by staining with DAPI for 10 min in an aluminium foil-covered box. Finally, the slides were covered with anti-fade mounting medium (P0128, Beyotime Biotechnology) for imaging.

### Statistical Analysis

All the data are presented as the mean ± SD. Student’s t-test or one-way ANOVA was used for the determination of statistical significance. The level of significance was defined as *p* < 0.05.

## Results

### The Characterization and Properties of the PLGA and PDA-PLGA Scaffold

The PDA coating adheres to the surface of the material but does not change the chemical properties of PLGA through the infrared spectra of the scaffolds (Supplementary FigureS1A). Through SEM observation, we found that the PDA coating changes the surface properties of the PLGA scaffold. Topographic stimulation caused by the increase in scaffold surface roughness may enhance cell adhesion and proliferation on the scaffold (Supplementary Figure S1B). As shown in Supplementary Figure S1C, the hydrophilicity of the PLGA scaffold before and after modification with the PDA coating was significantly different (*p* < 0.001), showing good hydrophilicity, and the tensile strength and elastic modulus of the PLGA scaffolds were significantly different before and after PDA coating modification (Supplementary Figures 1D,E). The results about degradation behaviour of the PDA-PLGA scaffold *in vitro* was show that the PDA coating improves the stability of the PLGA scaffold and can be used in more fields of medicine and materials.

### Effects of the Scaffolds on the Proliferation and Morphology of Various Cell Lines

**FIGURE 1:**
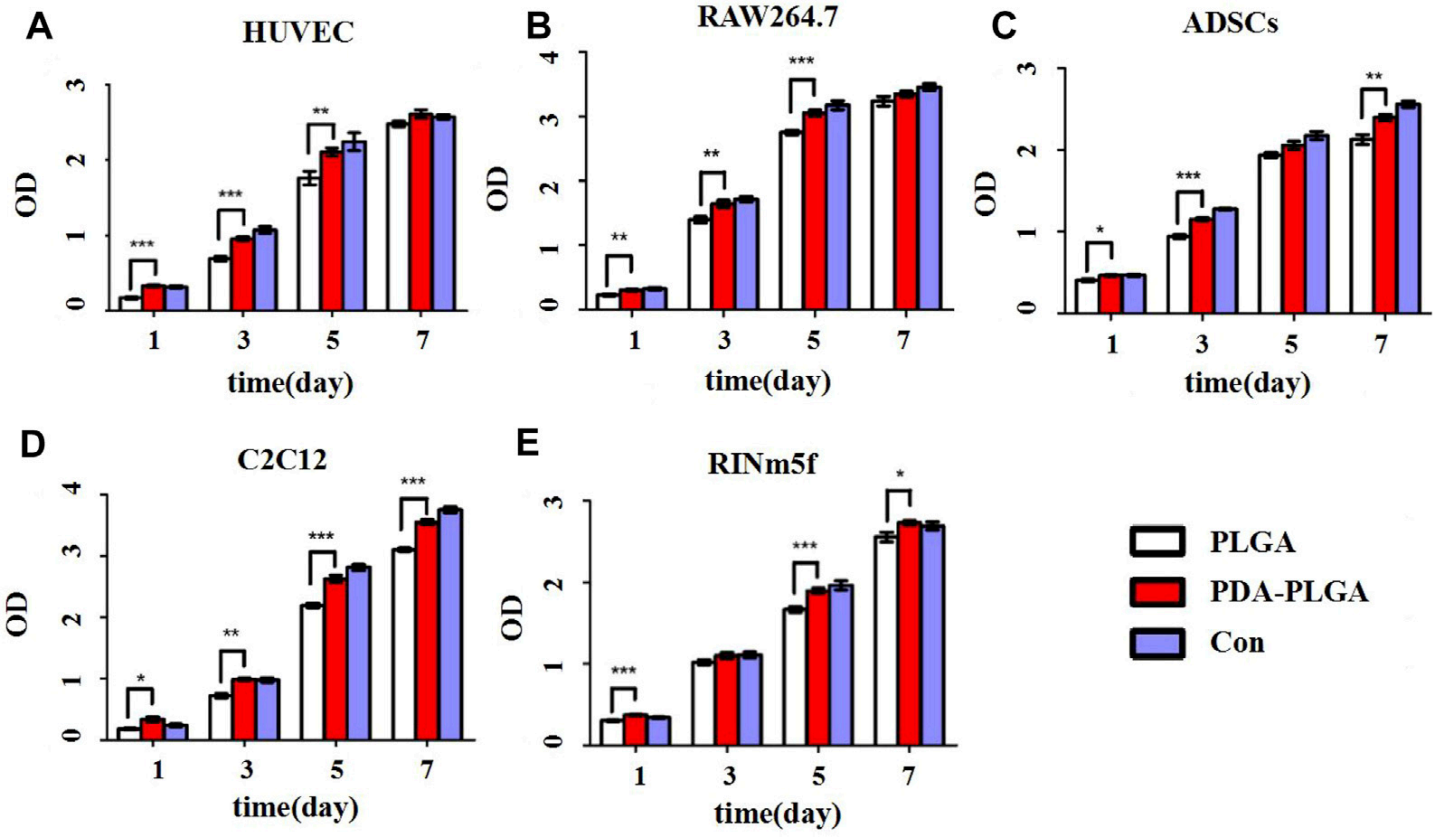
Proliferation of different cell types on the PDA-PLGA scaffold. **(A)** HUVECs. **(B)**: RAW264.7 cells. **(C)** ADSCs. **(D)** C2C12 cells. **(E)** RINm5f cells. Con: control group, PLGA: cell and PLGA scaffold coculture group, PDA-PLGA: cell and PDA-PLGA scaffold coculture group. *n* = 3, *p < 0.05, **p < 0.01 and ***p < 0.001 compared to the PLGA group.

**FIGURE 2:**
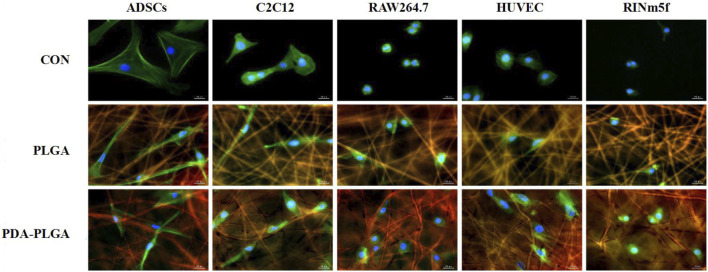
The adhesion morphology of different types of cells on the PDA-PLGA scaffold. Con: control group, PLGA: cell and PLGA scaffold coculture group, PDA-PLGA: cell and PDA-PLGA scaffold coculture group. Cells were stained for F-actin (green), nuclei (blue), and fibre scaffolds (red) with fluorescent dyes. Scale bars = 20 μm.

To evaluate the biosafety of our PDA-PLGA scaffold, we first tested its safety *in vitro*. The effects of the PDA-PLGA scaffold on the proliferation of HUVECs, RAW264.7 cells, ADSCs, C2C12 cells and RINm5f cells were detected by the MTT method. The results showed that the scaffold had no adverse effect on cell proliferation and growth when coincubated with different cells (Figure 1). To evaluate the effect of PDA-PLGA scaffold on cell morphology and adhesion, the adhesion morphology of different kinds of cells on PLGA cell scaffolds was observed by fluorescence microscopy. The results showed that the cells could adhere to the cell scaffold, and a slender pseudopod was observed extending into the 3D cell scaffold nanofibre structure. Cells attach to the surface through extended filamentous pseudopodia and tend to grow along polymer nanofibres on their surfaces (Figure 2).

### Effect of the PDA-PLGA Scaffold on Biochemical Indexes in the Serum and Tissue of Rats

**FIGURE 3:**
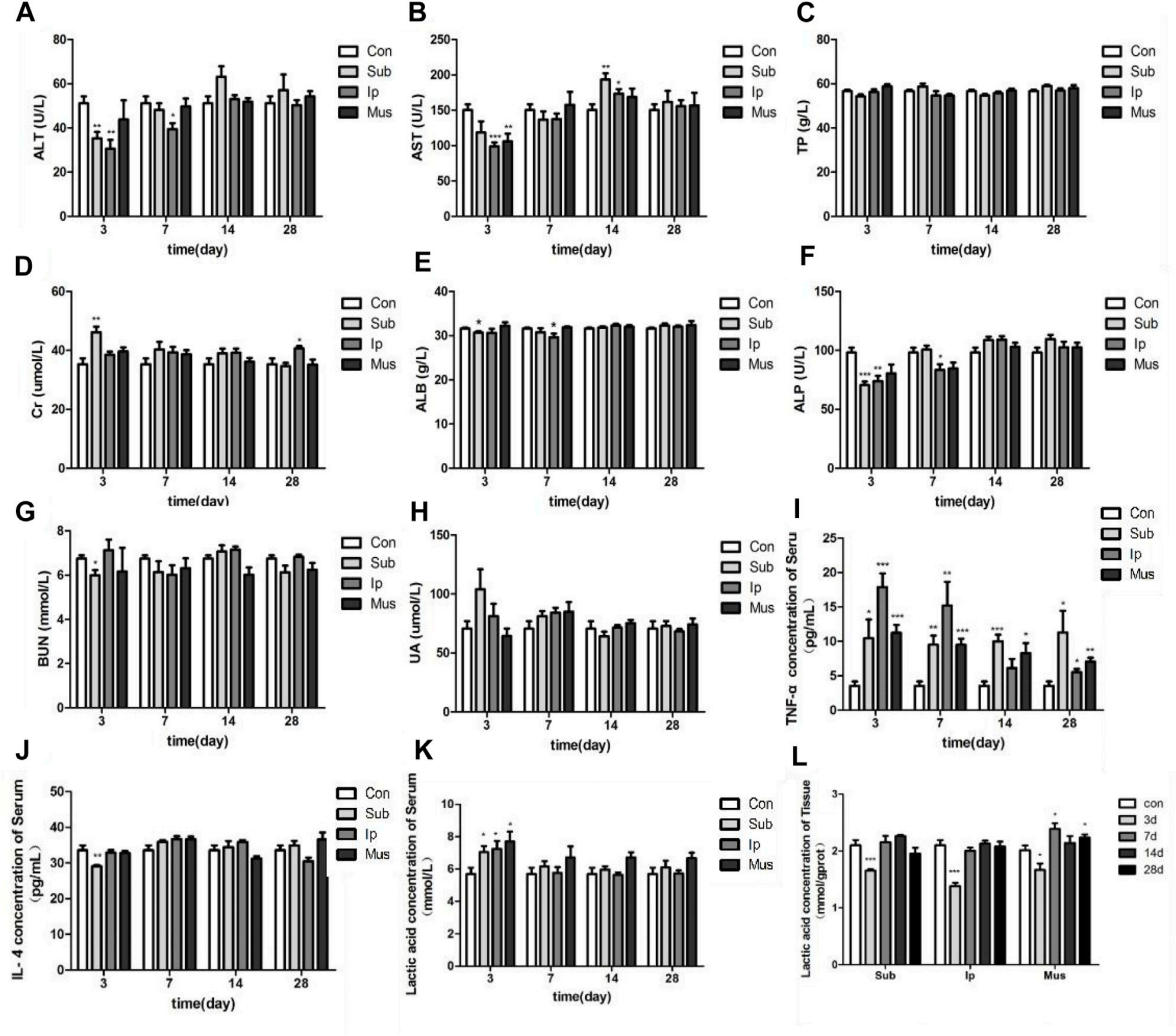
Changes in serum biochemical indexes and tissue lactic acid levels in rats at different locations and at various time points after PDA-PLGA transplantation. **(A)** ALT level. **(B)** AST level. **(C)** TP level. **(D)** Cr level. **(E)** ALB level. **(F)** ALP level. **(G)** BUN level. **(H)** UA level. **(I)** TNF-α level. **(J)** IL-4 level. **(K)** Blood lactic acid level. **(L)**: Tissue lactic acid level. Con: control group, Sub: subcutaneous transplantation group, Ip: intraperitoneally transplanted group, Mus: skeletal muscle transplantation group. *n* = 6, *p < 0.05, **p < 0.01 and ***p < 0.001 compared to the Con group.

After transplantation of the PDA-PLGA scaffold at three different sites, the rats recovered well, the incision healed well, and there was no infection (data not shown). As shown in Figure 3A–H, the AST, ALP, ALT, and other indexes fluctuated obviously in the early stage of transplantation. On the 3rd day, the contents of ALT, ALB, ALP, and BUN in the subcutaneous transplantation group decreased significantly, while the content of Cr increased in the subcutaneous transplantation group, while the ALT, AST, and ALP contents decreased in the intraperitoneal transplantation group. On the 7th day after transplantation, except for the decrease in ALT, ALB, and ALP contents in the intraperitoneal transplantation group, there was no difference in details between the other groups and the control group. On the 14th day of transplantation, except for the level of AST in the subcutaneous transplantation group and the intraperitoneal transplantation group, which increased, the other indexes were at the normal level. On the 28th day, the level of Cr in the intraperitoneal transplantation group increased, and all the indexes in the other groups were at the normal level. Except for the decrease in AST levels on the 3rd day in the skeletal muscle transplantation group, there was no significant difference in all indexes between the skeletal muscle transplantation group and the control group at other time points. The above results showed that the PDA-PLGA scaffold affected all the indexes of the rat body at the initial stage of transplantation but basically returned to normal after 14 days, and transplantation in the skeletal muscle site had the least effect on the liver function and kidney function of rats.

Compared with the Con group, the level of TNF-α in the transplanted rats increased significantly, indicating that the transplanted fibrous scaffold as a foreign body caused a certain inflammatory response. Although the content of TNF-α decreased with the extension of time, it was still higher than the normal level, while the content of IL-4 only decreased in the early stage of the subcutaneous transplantation group, and there was no significant difference between the other time points and the control group (Figures 3I,J). The contents of lactic acid in the serum and tissue of rats were detected at each time point after transplantation. As shown in Figures 3K,L, the content of lactic acid in serum increased in each group on the 3rd day of transplantation but decreased significantly compared with the control group in tissue. Then, the content of serum lactic acid gradually returned to normal. The content of lactic acid in the skeletal muscle transplantation group increased on the 7th and 28th days, which may be due to the degradation of scaffold materials and the increase in lactic acid produced by muscle.

### Effects of the PDA-PLGA Scaffold on Tissues and Organs of Rats

**FIGURE 4:**
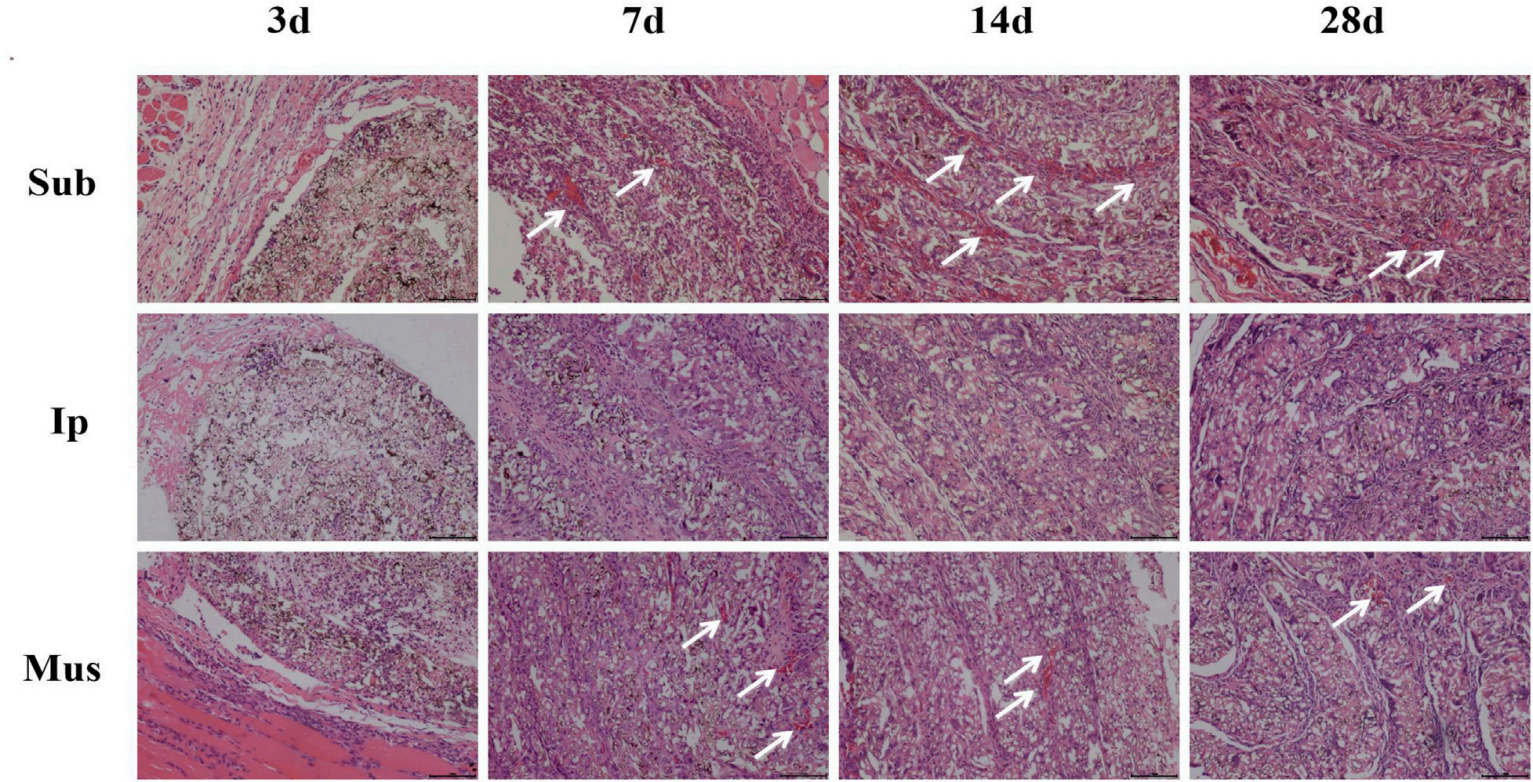
HE staining of the PDA-PLGA scaffold at the rat transplantation site (100×). Sub: subcutaneous transplantation group, Ip: intraperitoneally transplanted group, Mus: skeletal muscle transplantation group.

**FIGURE 5:**
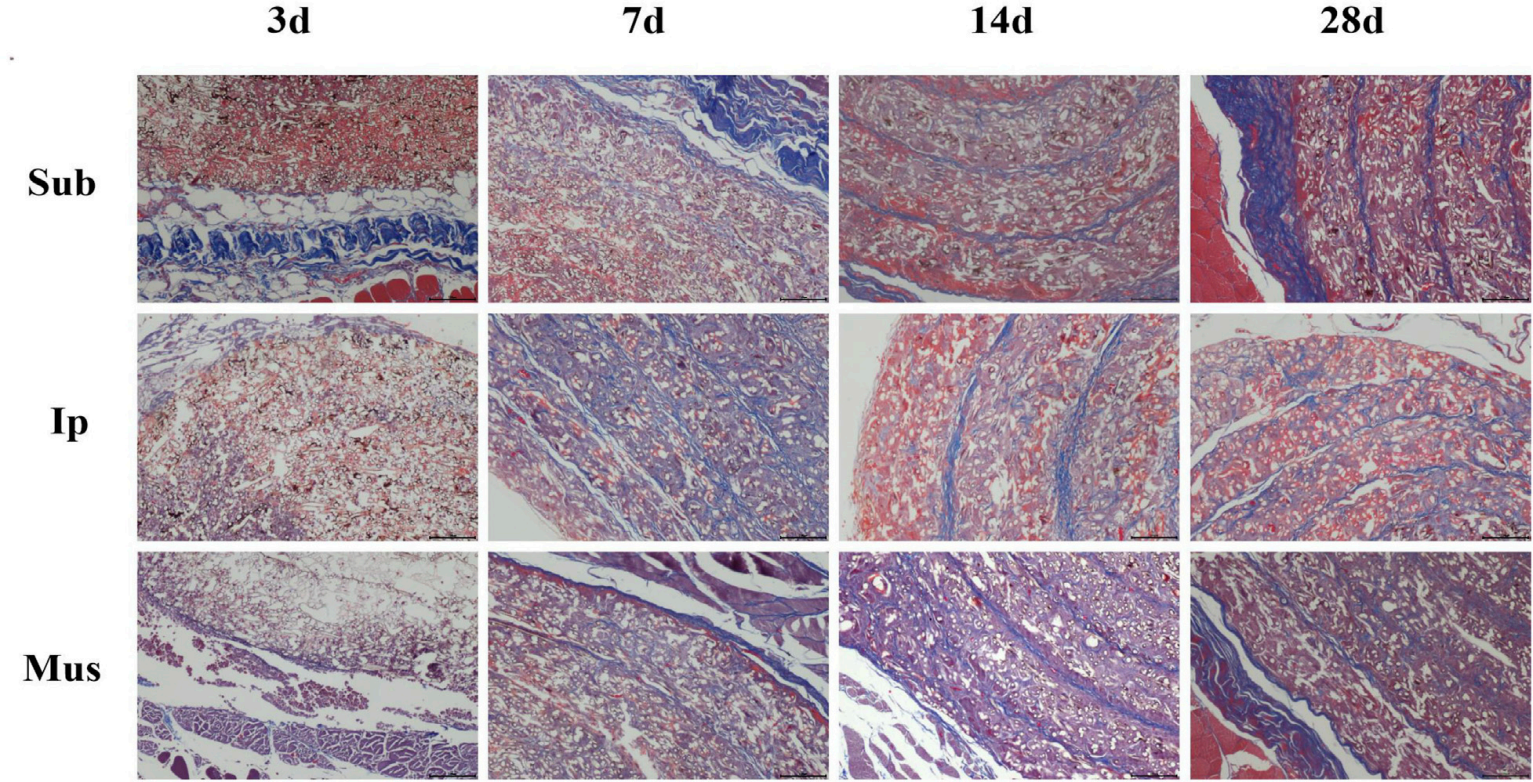
Masson trichrome staining of the PDA-PLGA scaffold at the rat transplantation site (100×). Sub: subcutaneous transplantation group, Ip: intraperitoneally transplanted group, Mus: skeletal muscle transplantation group.

**FIGURE 6:**
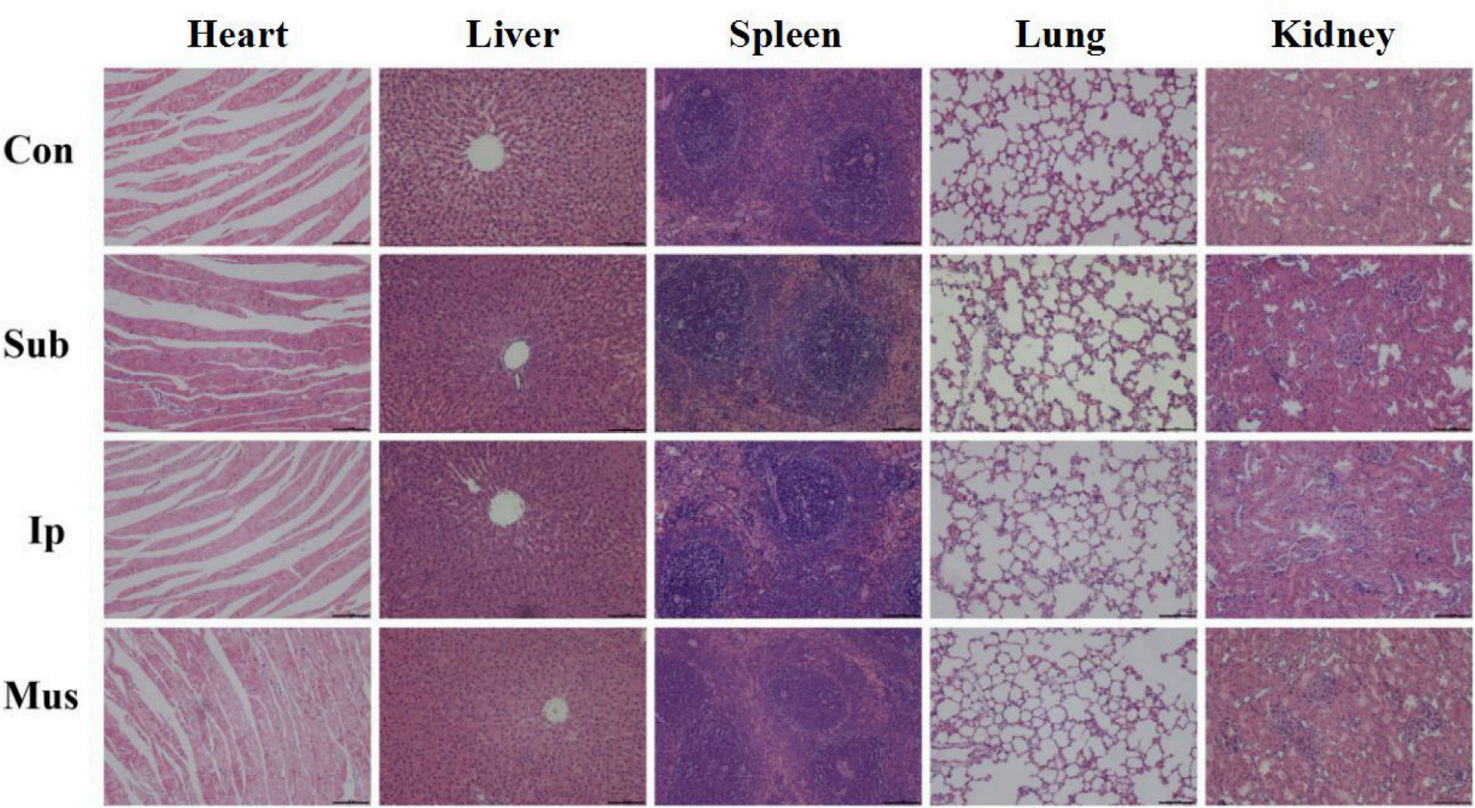
Representative images of H&E staining of the main organs, including the heart, liver, spleen, lungs and kidneys, from rats after transplantation for 28 days. The scale bars represent 100 μm.

On the 3rd, 7th, 14th and 28th days, some rats were killed, the main organs were observed with the naked eye, and no changes, such as hyperaemia and necrosis, were found. As shown in Figures 4, 5, obvious cell infiltration and orientation formed by material curling could be observed at the transplantation site after transplantation. Inflammatory cells were mainly attached to the surface of the material, and cell affinity was conducive to blood vessel growth. Red blood cells were found in the subcutaneous transplantation group and skeletal muscle transplantation group from the 7th day, and small blood vessels gradually formed, which was beneficial to the survival of the graft. Masson staining showed that the trend of cell infiltration and neovascularization was similar to that of HE staining, and the collagen fibres were mainly concentrated on the surface of the material and gradually increased with time. As shown in Figure 6, the main organs of the rats 28 days after transplantation were compared with those of the control group, and there was no obvious abnormality. In summary, no pathological changes caused by the PDA-PLGA scaffold was found by histopathological examination and the PDA-PLGA scaffold had good biological safety.

### Effects of the PDA-PLGA Scaffold Loaded With RINm5f Cells on Insulin Secretion

**FIGURE 7:**
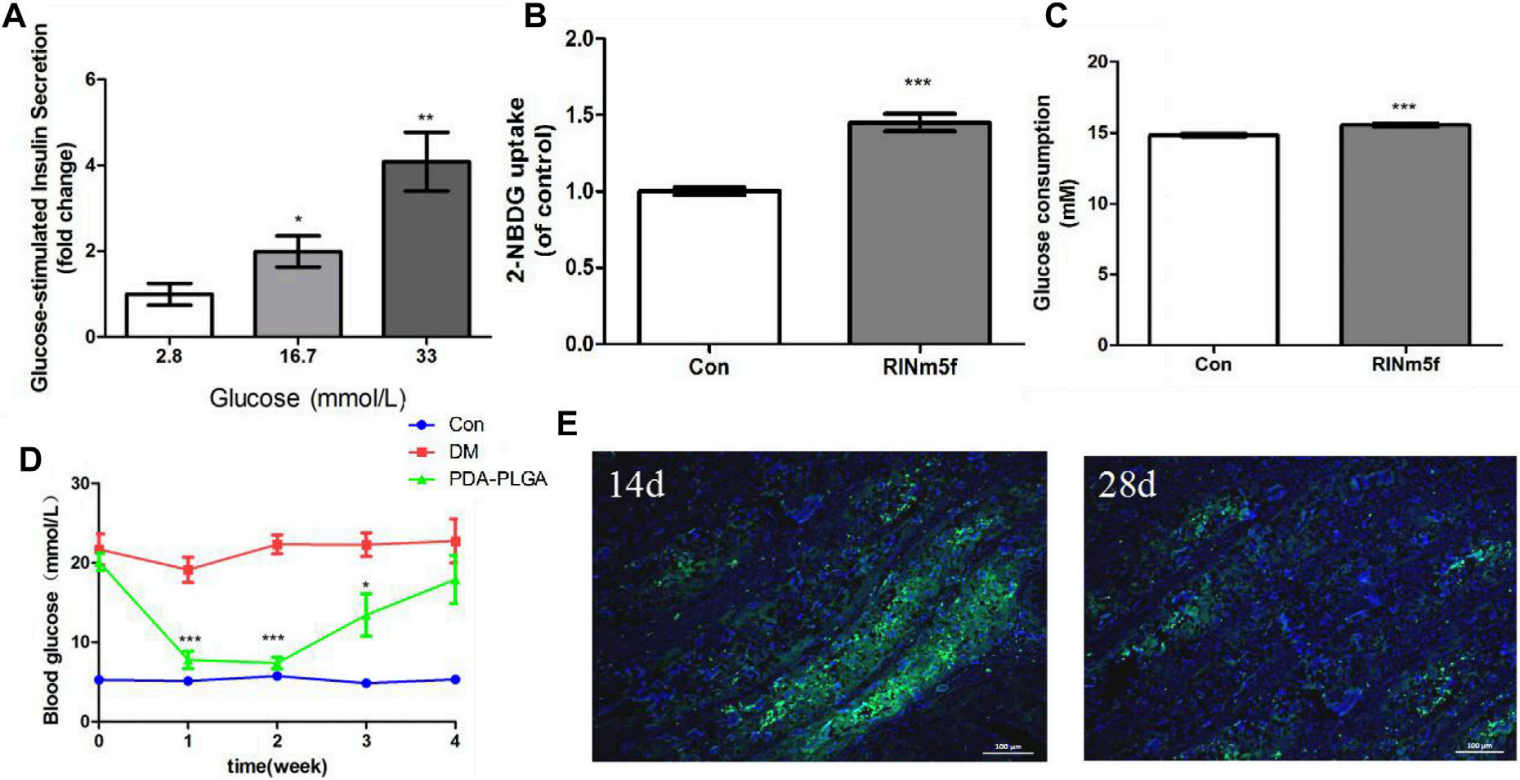
Therapeutic effect of the PDA-PLGA scaffold with islet cell skeletal muscle transplantation on the treatment of type 1 diabetic rats. **(A)**: Insulin secretion in RINm5f cells seeded onto the PDA-PLGA scaffolds stimulated by glucose, *n* = 3, *p < 0.05 and **p < 0.01 compared to the corresponding basal group with 2.8 mM glucose. **(B,C)**: Effect of RINm5f cell supernatant on skeletal muscle glucose uptake and consumption. Con: control group, RINm5f: RINm5f cell supernatant treatment group, *n* = 3, ***p < 0.001 compared to the Con group. **(D)**: Fasting blood glucose levels of rats after transplantation of RINm5f cells and PDA-PLGA cell scaffold complex, Con: control group, DM: diabetes model group, PDA-PLGA: islet cell transplantation treatment group, *n* = 6, ***p < 0.001 and *p < 0.05 compared to the DM group. **(E)**: RINm5f and PDA-PLGA cell scaffold complex skeletal muscle transplantation insulin immunofluorescence results at different times. Cells were stained for nuclei (blue) and insulin (green) with fluorescent dyes. Scale bars = 100 μm.

The insulin secretion of islet cells under different concentrations of glucose stimulation was detected by GSIS when islet cells were cocultured with the PDA-PLGA scaffold. As shown in Figure 7A, when RINm5f cells were cocultured with the PDA-PLGA scaffold, the secretion function was normal, and the amount of insulin secretion increased with increasing glucose concentration, showing an obvious dose-effect relationship. The results show that the PDA-PLGA scaffold had no effect on the secretory function of islet cells and can be used as a carrier for islet cell transplantation.

### Effects of the PDA-PLGA Scaffold Loaded With RINm5f Cells on Glucose Uptake and Consumption by C2C12 Cells

In view of the fact that skeletal muscle is the key target organ of glucose metabolism, we chose skeletal muscle as the transplantation site of islet cells. To verify whether insulin secreted by RINm5f cells in PDA-PLGA scaffold can promote glucose uptake and glucose consumption in skeletal muscle cells and achieve hypoglycaemia, we coincubated C2C12 cells with the supernatant of RINm5f cells to detect their effects on glucose consumption and glucose uptake in skeletal muscle C2C12 cells. The results of cellular glucose uptake and glucose consumption are shown in Figures 7B,C. After treatment with RINm5f cell supernatant, the basal metabolic glucose uptake of skeletal muscle cells was significantly higher than that of the control group (*p* < 0.001), and the glucose consumption increased significantly (*p* < 0.001). The results showed that insulin secreted by RINm5f can enhance skeletal muscle glucose uptake and consumption. At the cellular level, islet cell transplantation in skeletal muscle is expected to reduce blood glucose and achieve the purpose of treating diabetes.

### Therapeutic Effect of PDA-PLGA Scaffold Skeletal Muscle Transplantation Loaded With Islet Cells on Type 1 Diabetic Rats

Compared with the control group, the fasting blood glucose of rats in the treatment group and model group increased significantly after STZ (*p* < 0.001), and the fasting blood glucose was ≥11.1 mmoL. One week after the transplantation of islet cells and the PDA-PLGA scaffold complex, the blood glucose of the treatment group was significantly lower than that of the model group (*p* < 0.001). It was proven that the transplanted islet cells played a role, secreted insulin and decreased the blood glucose of rats, but approximately 4 weeks after transplantation, the blood glucose of the treatment group increased, which was no different from that of the model group. It is speculated that the inflammatory reaction may be caused by foreign bodies. The complex microenvironment in the body led to the gradual death of islet cells and the disappearance of the hypoglycaemic effect (Figure 7D).

To verify the therapeutic effect of RINm5f cells and the PDA-PLGA scaffold complex on type I diabetic rats, fixed sections of muscle and grafts were obtained for insulin immunofluorescence detection, and the survival and insulin secretion of RINm5f cells were observed. The results of insulin immunofluorescence are shown in Figure 7E. In the treatment group, insulin green fluorescence was obvious on the 14th day, insulin secretion was large, and the hypoglycaemic effect was significant; on the 28th day, insulin green fluorescence was obviously weakened, and islet cells basically died. In summary, the transplantation of islet cells in skeletal muscle with the PDA-PLGA scaffold has a certain therapeutic effect on type I diabetes, but the transplanted islet cells are still allotransplanted, which is easily affected by rejection and mechanical pressure caused by skeletal muscle movement, resulting in cell death, so they still need to be further improved.

## Discussion

The functional damage of islet β cells is the core link in the pathogenesis of diabetes. The regeneration and transplantation of islet β cells and the reconstruction of insulin secretion function have become new strategies for curing diabetes, but the donor and site of transplantation and the selection of transplantation carriers are the key to this strategy. In this study, PDA-PLGA scaffold was prepared by electrospinning and polydopamine coating techniques, and their safety was evaluated by coculture of cells with scaffolds and *in vivo* transplantation of PDA-PLGA scaffold at different parts. On this basis, islet cells were inoculated into the PDA-PLGA scaffold to construct a cell-scaffold complex for *in vivo* transplantation in diabetic rats. After a preliminary study of its therapeutic effect on diabetes, the following important findings were obtained: 1) The PDA-PLGA scaffold had better biological safety, in which skeletal muscle stent transplantation has less impact on various indexes of the body and is conducive to angiogenesis, which also shows that skeletal muscle as a transplant site has a certain development potential. 2) The transplantation of islet cells in the skeletal muscle of diabetic rats with the PDA-PLGA scaffold could treat diabetes and lasts for approximately 3 weeks.

Biological scaffolds play a fundamental role in tissue engineering, which can provide mechanical and structural support for cell adhesion and tissue development, act as extracellular matrix, and create an environment for cell proliferation and differentiation (Hendow et al., 2016). As the main body of scaffolds, polymer materials such as PLGA, which have been used in the study of some diseases (Lin et al., 2014; Niu et al., 2014; Uma Maheshwari et al., 2014; Zheng et al., 2014; Sidney et al., 2015; Ebrahimi et al., 2016). Qian et al. (Qian et al., 2016) found that the hydrophilicity and mechanical properties of PLGA/PCL blended fibre scaffolds modified by PDA coating were significantly improved. In the experiment, we prepared PLGA fibre scaffold and treated with PDA coating. The results show that after PDA coating, the water contact angle of the scaffold decreases obviously, the hydrophilicity increases, the elastic modulus and tensile strength increase significantly, and the degradation rate can be slowed down, which is basically consistent with the conclusions of other researchers.

Biosafety is an important prerequisite for the clinical application of scaffolds. Rim et al. (Rim et al., 2012) showed that the surface modification of a PLA scaffold with a PDA coating could enhance the adhesion of mesenchymal stem cells to the fibre surface. Jeong et al. (Wang et al., 2011) used PDA to modify the surface of polymethyl methacrylate (PMMA) and found that it improved the biological binding of the material and promoted the proliferation of corneal epithelial cells and corneal stromal cells. We selected inflammatory cells, vascular endothelial cells, skeletal muscle cells, islet cells, adipose mesenchymal stem cells and other different cell lines for *in vitro* experiments, and the study shows that the cells can adhere to the fibre surface of the stent well. At present, research on PDA is mainly focused on the cellular level, but there are few experimental studies on *in vivo* transplantation, and there are no studies evaluating its safety *in vivo*. New nanomedicine strategy shown to be effective in improving tissue inflammation in diseases (Shi et al., 2022). We selected three different sites of rats to evaluate the safety of different transplantation sites. By comparison, skeletal muscle transplantation had the least effect on serum biochemical indexes, although the transplanted PDA-PLGA scaffold caused certain inflammatory reactions as a foreign body. The level of TNF-α increased, but with the extension of time, the level of inflammatory factors decreased gradually. Obvious cell infiltration and collagen accumulation could be found on the surface of the transplanted materials by HE and Masson staining. Angiogenesis could be found in the subcutaneous transplantation group and skeletal muscle transplantation group from the 7th day. Early angiogenesis was important for the survival of the grafts. The sections of the main organs of each group were stained with HE, and compared with the control group, no significant difference was found. In summary, the PDA-PLGA scaffold prepared in this study showed good biological safety in preclinical *in vivo* and *in vitro* experiments. Skeletal muscle as a stent transplantation site has less impact on various indicators of the body and is conducive to angiogenesis, so it may be a good site for cell transplantation.

Islet cell transplantation, as a promising alternative to total pancreatic transplantation, has been greatly developed in the past few years. However, this therapy also faces a number of challenges: for example, the function of the transplanted islet cells continues to decline due to the maladaptive nature of the implantation site (Brusko et al., 2021). A major technical obstacle in the cell transplantation process is the difficulty of islet β-cells surviving in the patient. Some studies have pointed out that more than half of the injected islets are acutely damaged after transplantation, liver islet injections can also lead to islet cell death and substantial loss due to severe inflammatory reactions and inadequate islet haemodialysis (Moberg et al., 2002; Augsornworawat et al., 2020). However, unlike whole-tissue transplantation, the location selection and implantation parameters of β-cell transplantation are highly flexible. Therefore, in order to improve the survival rate after β-cell transplantation, researchers need breakthroughs in the following two main directions: first, to find more suitable transplantation sites. Second, material-based adjuvants.

Studies have shown that in the field of islet transplantation, sodium alginate has been used for islet cell transplantation through microcapsules and giant capsules (Dang et al., 2013; Khanna et al., 2013; Chen et al., 2015; Llacua et al., 2016). In addition, Kim JS et al. (Kim et al., 2019) implanted a flake PLGA scaffold coated with hypo-MSCs (mesenchymal stem cells cultured in hypoxia) subcutaneously with islet cells in mice, which also confirmed its therapeutic effect on diabetes. At present, there is no study on the use of PDA-PLGA scaffold in the treatment of islet cell transplantation. To explore whether the PDA-PLGA scaffold can be used as a carrier to carry islet cells for *in vivo* transplantation for the treatment of diabetes, we cocultured PDA-PLGA scaffold with RINm5f cells to construct the transplantation complex and, according to previous experimental results, determined the site of skeletal muscle as the treatment site of diabetic cell transplantation. Some studies have shown that skeletal muscle, as a key target organ for glucose utilization, shows high blood oxygen tension, meets the requirements of islet cells for the microenvironment and is an ideal islet implantation site (Wolf-Van Buerck et al., 2015). In this study, we found that the PDA-PLGA scaffold had no effect on islet cell secretion, and could promote glucose consumption and glucose uptake in C2C12. At the animal level, the cell-scaffold complex was transplanting into the skeletal muscle of diabetic rats. The results showed that the fasting blood glucose of the rats in the transplantation group decreased significantly, and the therapeutic effect disappeared on the 28th day after transplantation. It is speculated that the transplanted islet cells are affected by rejection, tissue mechanical pressure and fibrosis, resulting in cell death gradually, so it still needs to be improved to further prolong the cell survival time. We will try to combine scaffolds with hydrogel, matrix glue and other materials to enhance the mechanical strength of the scaffold, reduce the effect of inflammatory cell affinity on the growth of islet cells, and prolong the survival time of islet cells.

## Conclusion

In this study, we prepared polydopamine-coated nanofibre scaffolds (PDA-PLGA). In the follow-up experiment, we cocultured islet cells with scaffolds and transplanted them into the skeletal muscle of diabetic rats. The results showed that the skeletal muscle as the transplantation site for PDA-PLGA cell scaffold could reduce the blood glucose in diabetic rats successfully and lasted for approximately 3 weeks, which further indicated the skeletal muscle transplantation site was a new choice for islet cell transplantation in the future.

## Data Availability

The original contributions presented in the study are included in the article/Supplementary Material, further inquiries can be directed to the corresponding authors.

## Abbreviations

PLGA, Lactic acid-glycolic acid copolymer; PDA-PLGA, Lactic acid-glycolic acid copolymer scaffold was modified by polydopamine coating; STZ, Streptozotocin; AST, Aspartate aminotransferase; ALT, Alanine aminotransferase; TP, Total Protein; ALB, Serum albumin; ALP, Alkaline phosphatase; BUN, Blood urea nitrogen; Cr, Creatinine; UA, Uric acid; TNF-α, Tumor necrosis factor-α; IL-4, Interleukin.
