# Supplementary material for: Study on the Effect of PDA-PLGA Scaffold Loaded With Islet Cells for Skeletal Muscle Transplantation in the Treatment of Diabetes
Source: Front Bioeng Biotechnol. 2022 Jun 30;10:927348. doi: 10.3389/fbioe.2022.927348 (PMC9280155; doi:10.3389/fbioe.2022.927348)
Supplement: Supplementary file 1 [file Presentation1.PPTX]

## Slide 1
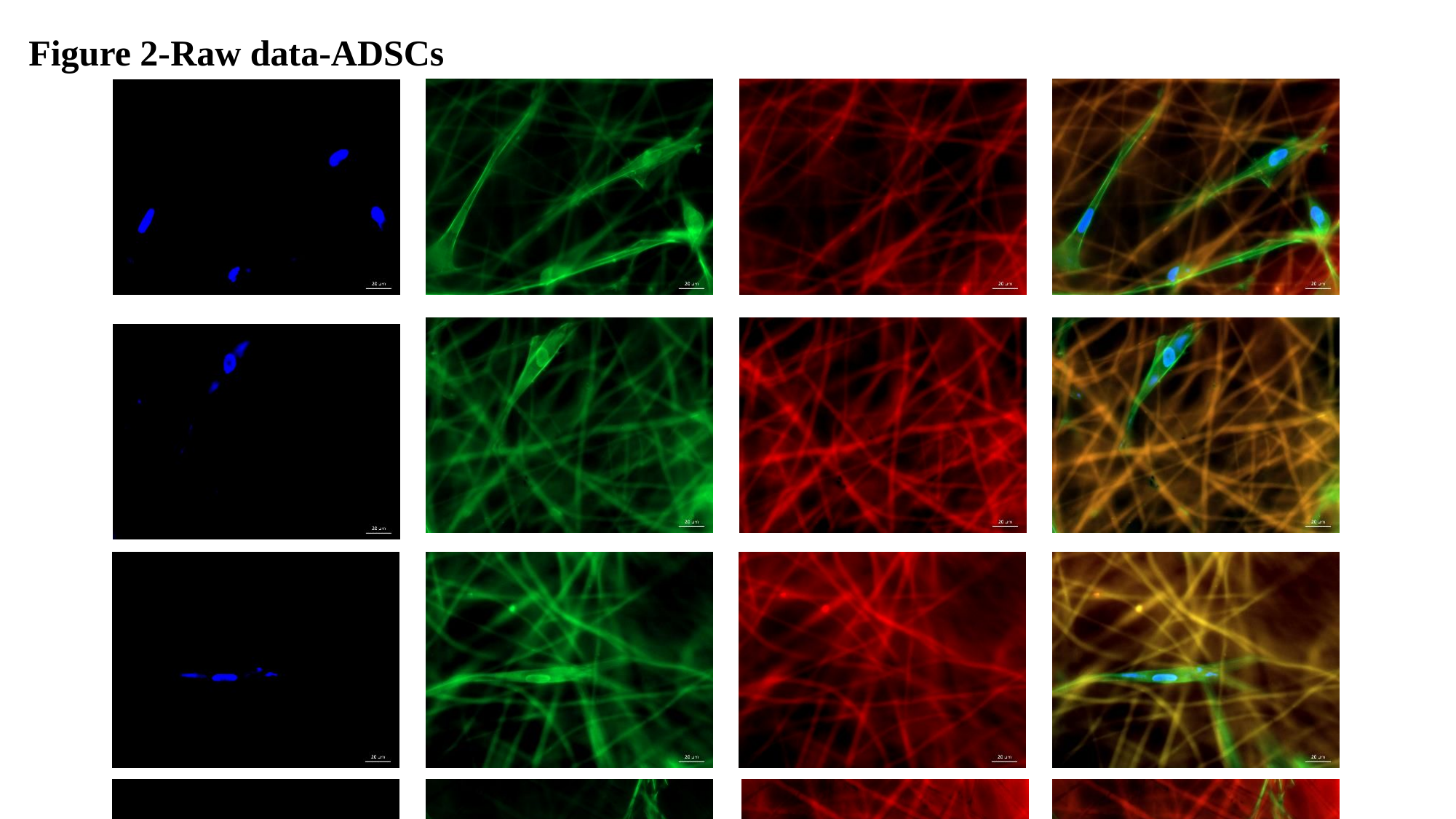

Figure 2-Raw data-ADSCs

## Slide 2
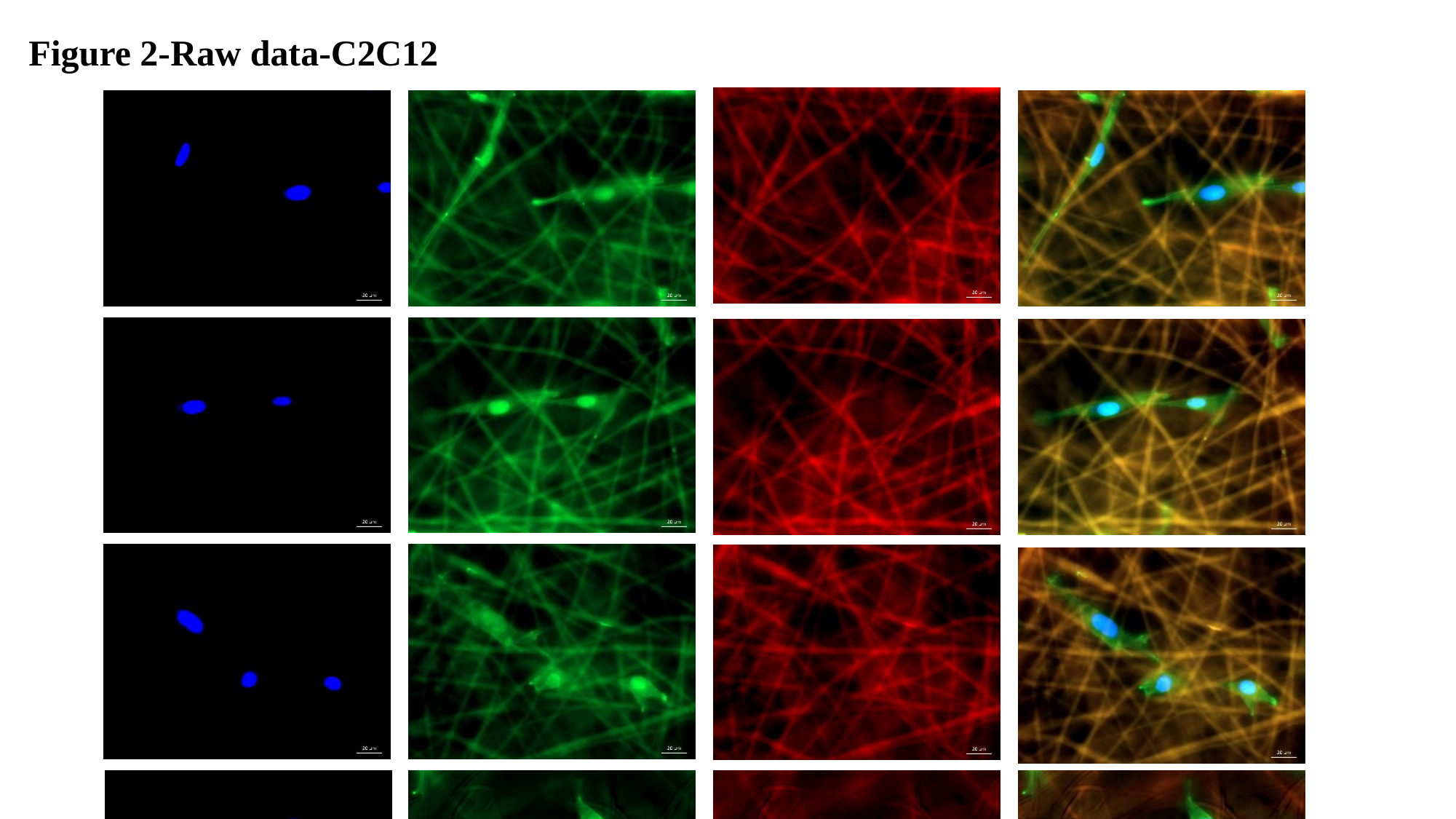

Figure 2-Raw data-C2C12

## Slide 3
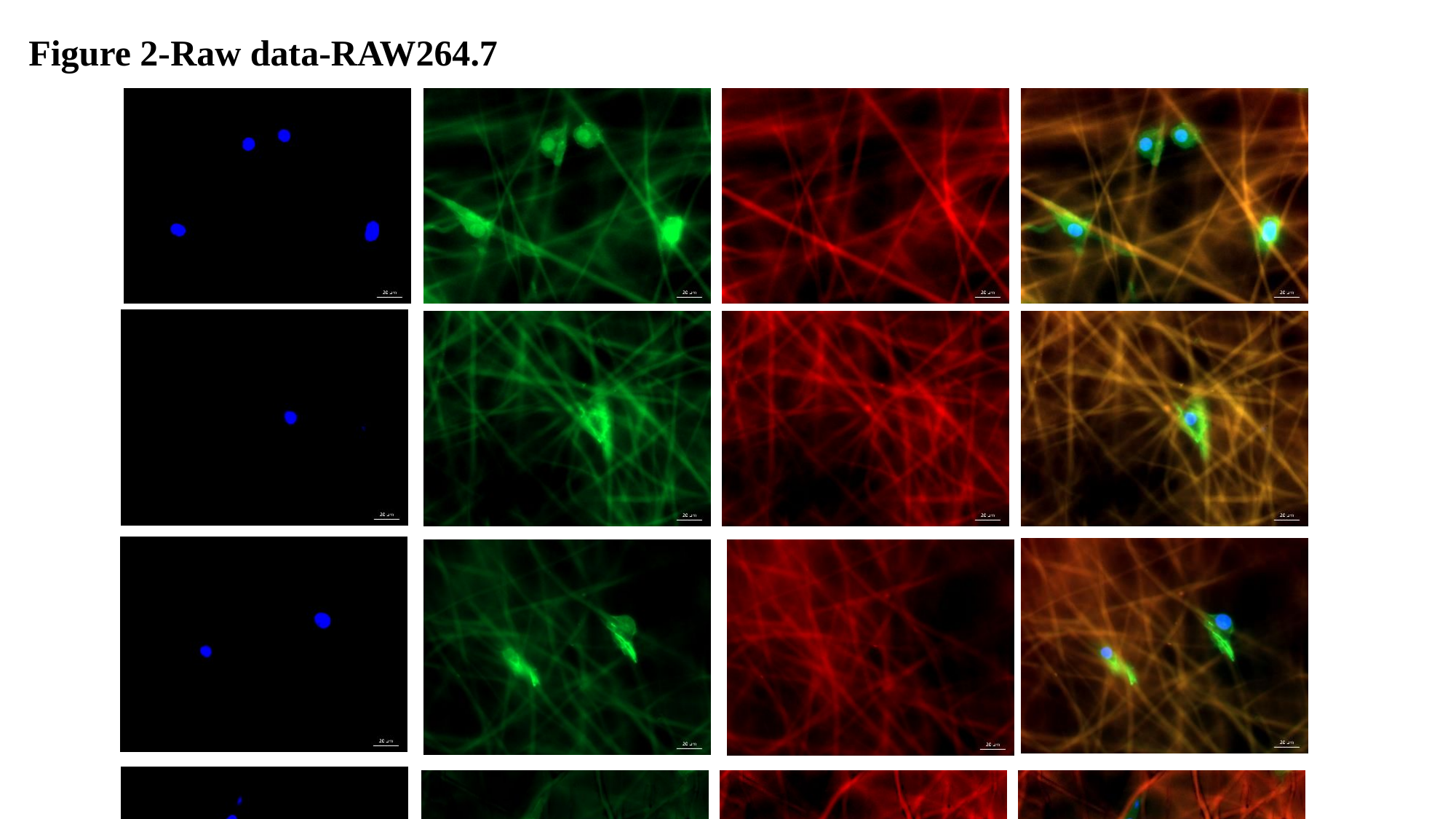

Figure 2-Raw data-RAW264.7

## Slide 4
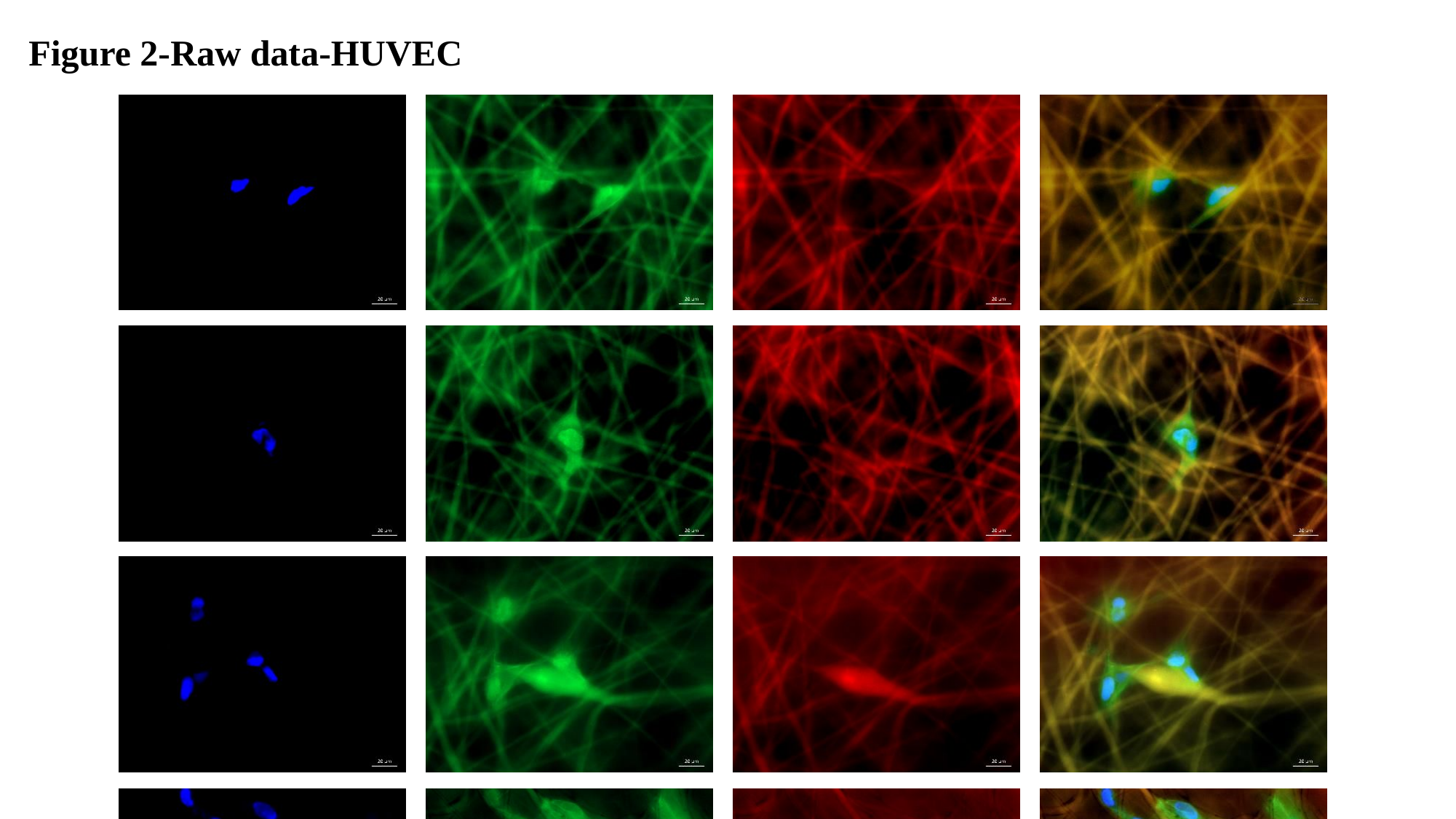

Figure 2-Raw data-HUVEC

## Slide 5
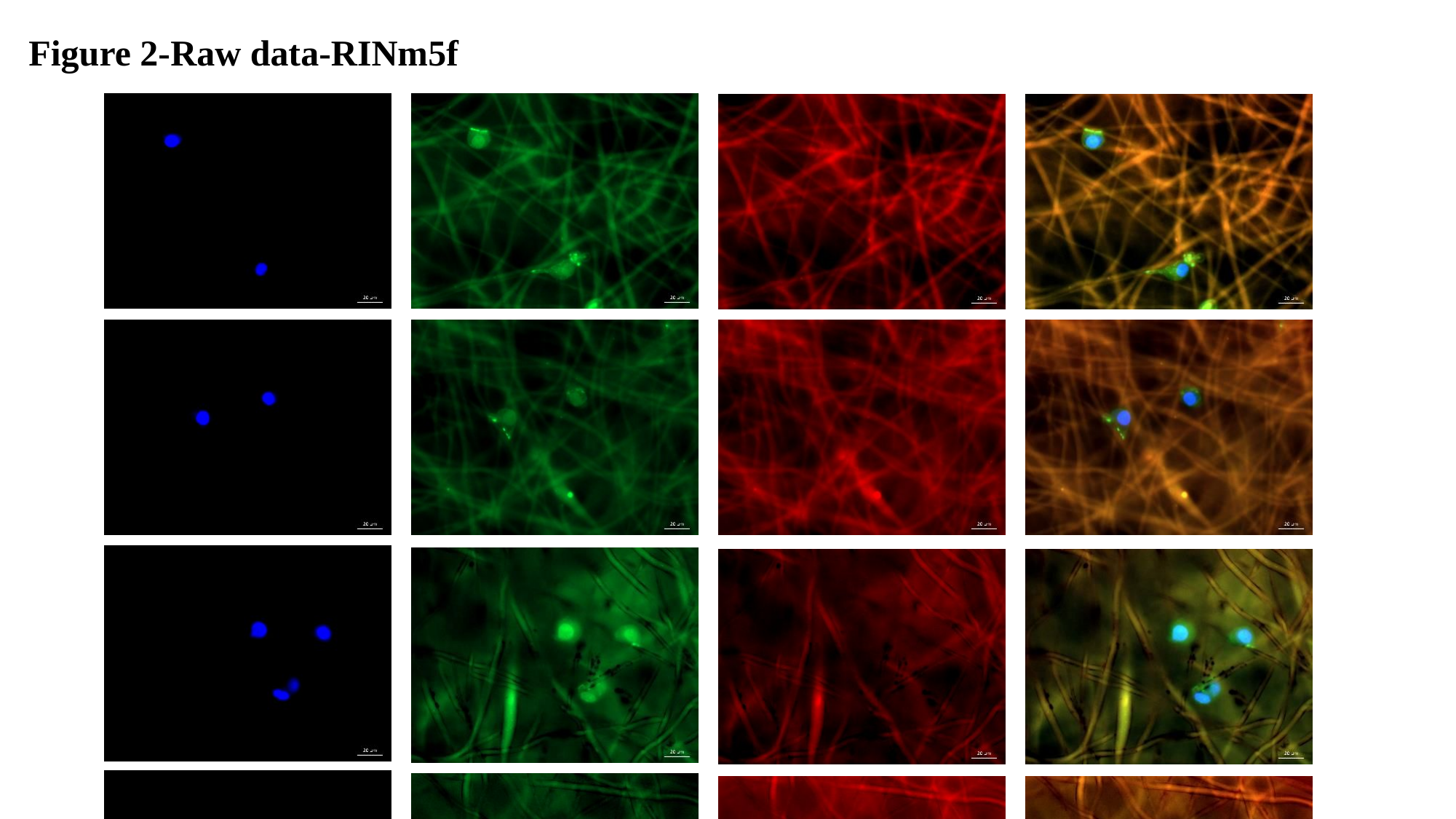

Figure 2-Raw data-RINm5f

## Slide 6
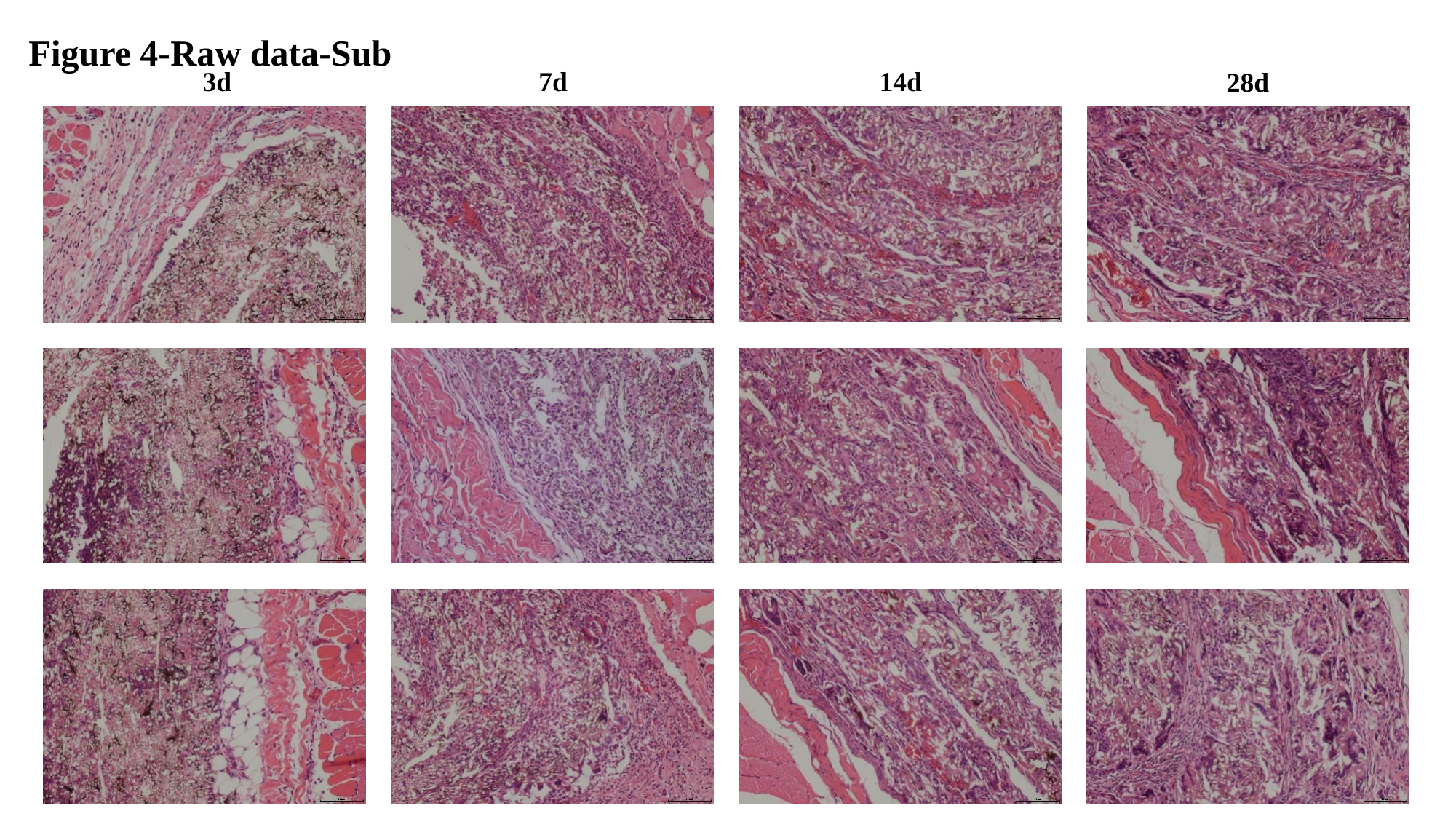

Figure 4-Raw data-Sub
3d
7d
14d
28d

## Slide 7
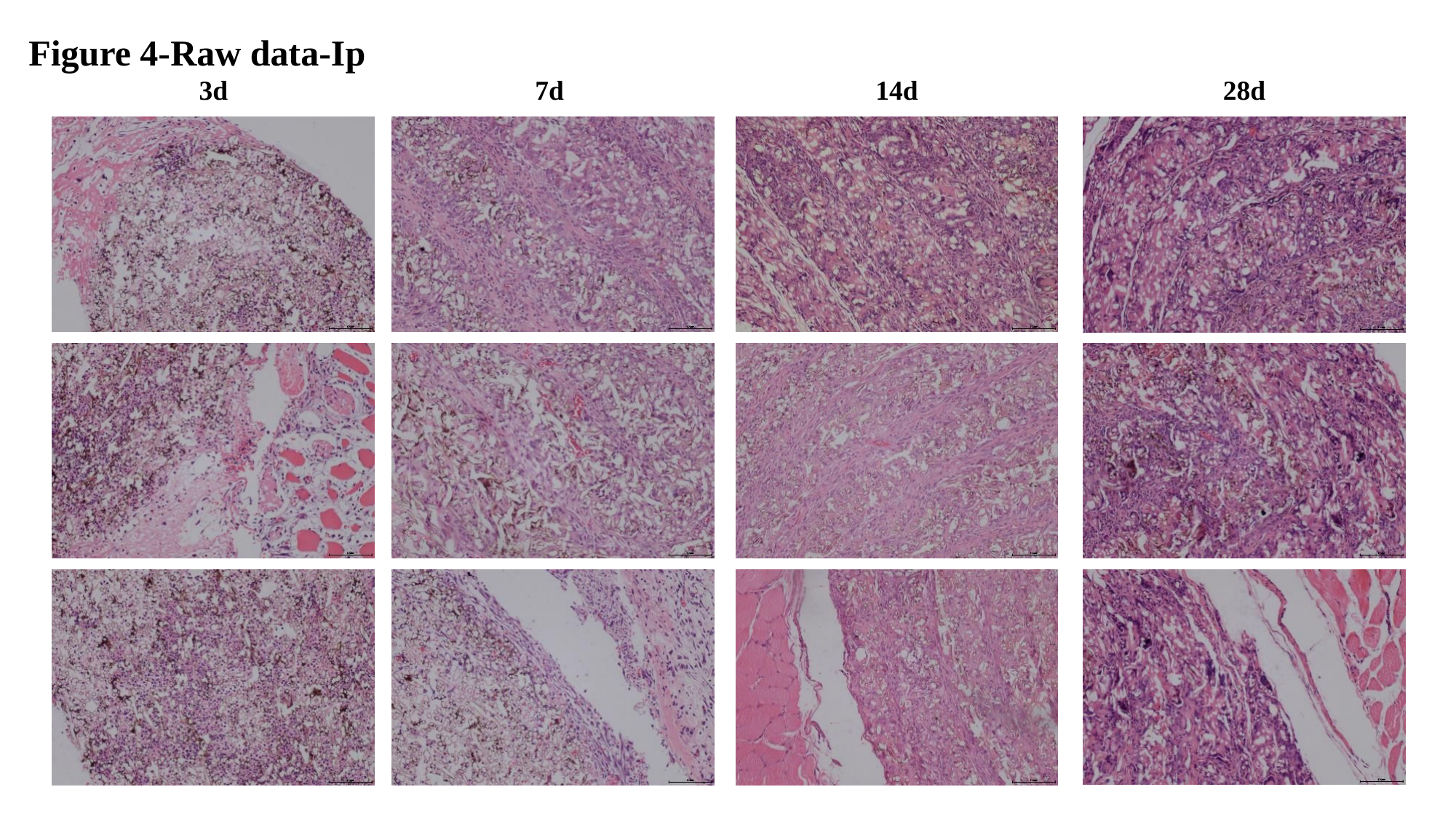

Figure 4-Raw data-Ip
3d
7d
14d
28d

## Slide 8
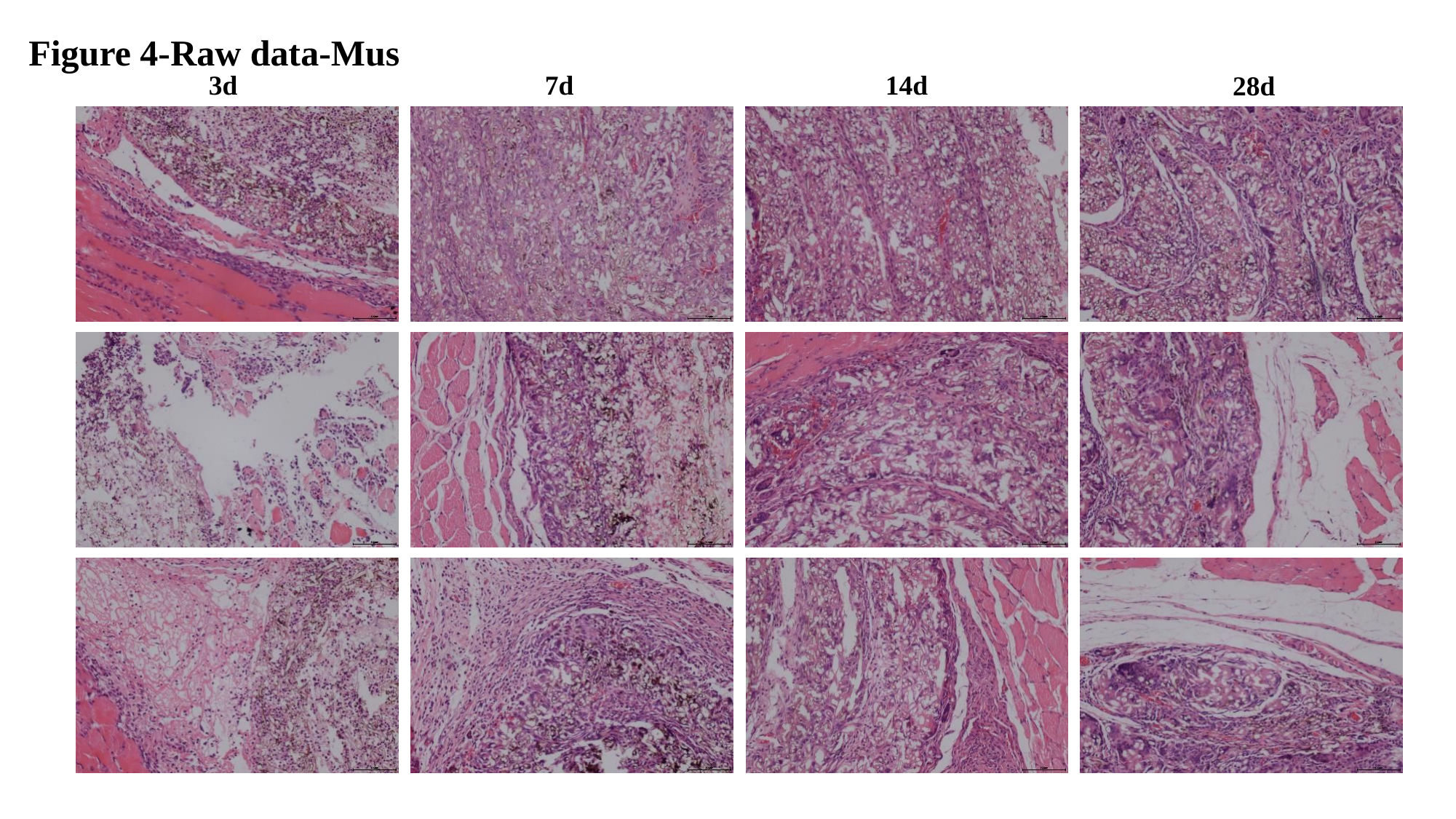

Figure 4-Raw data-Mus
3d
7d
14d
28d

## Slide 9
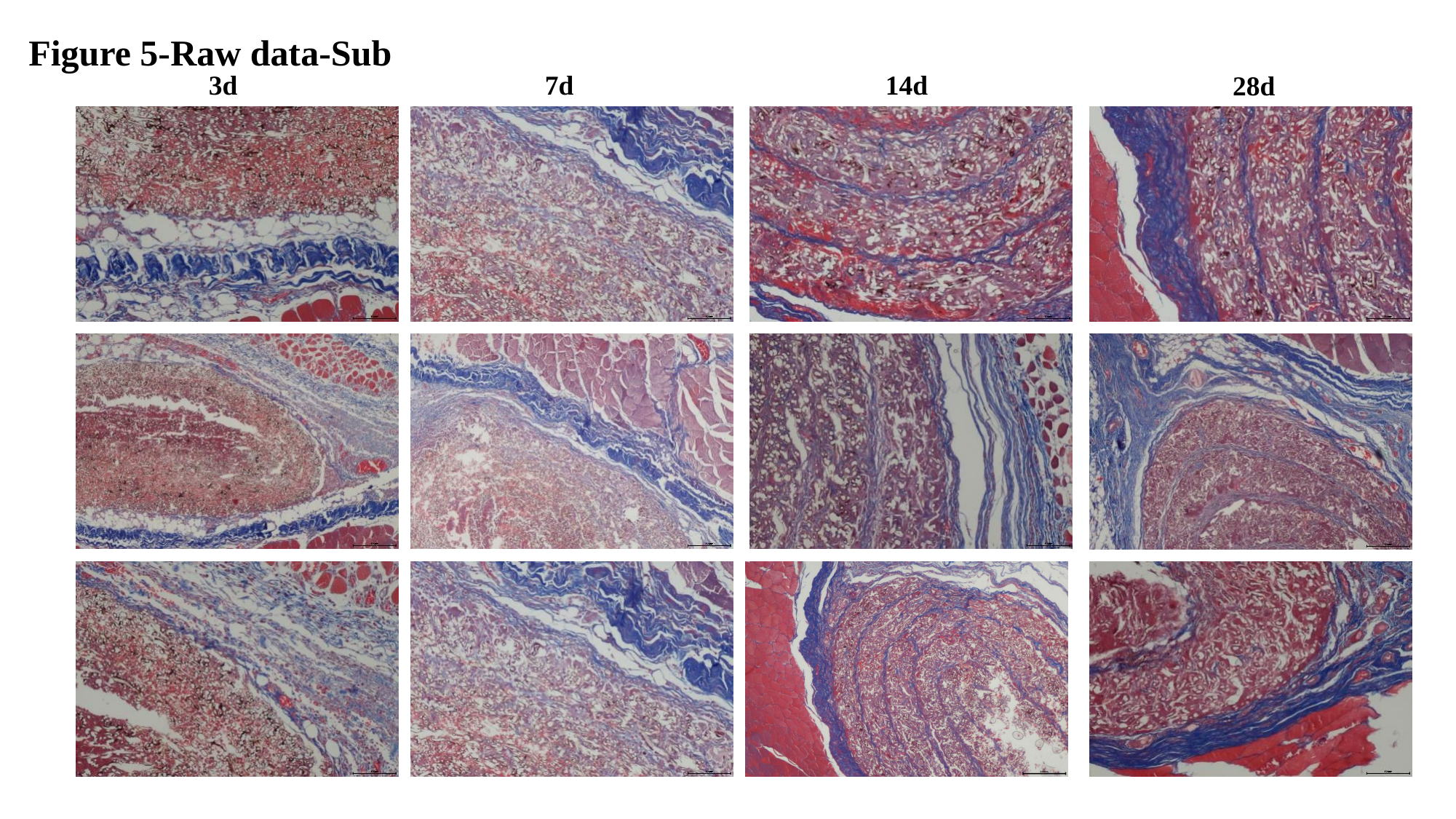

Figure 5-Raw data-Sub
3d
7d
14d
28d

## Slide 10
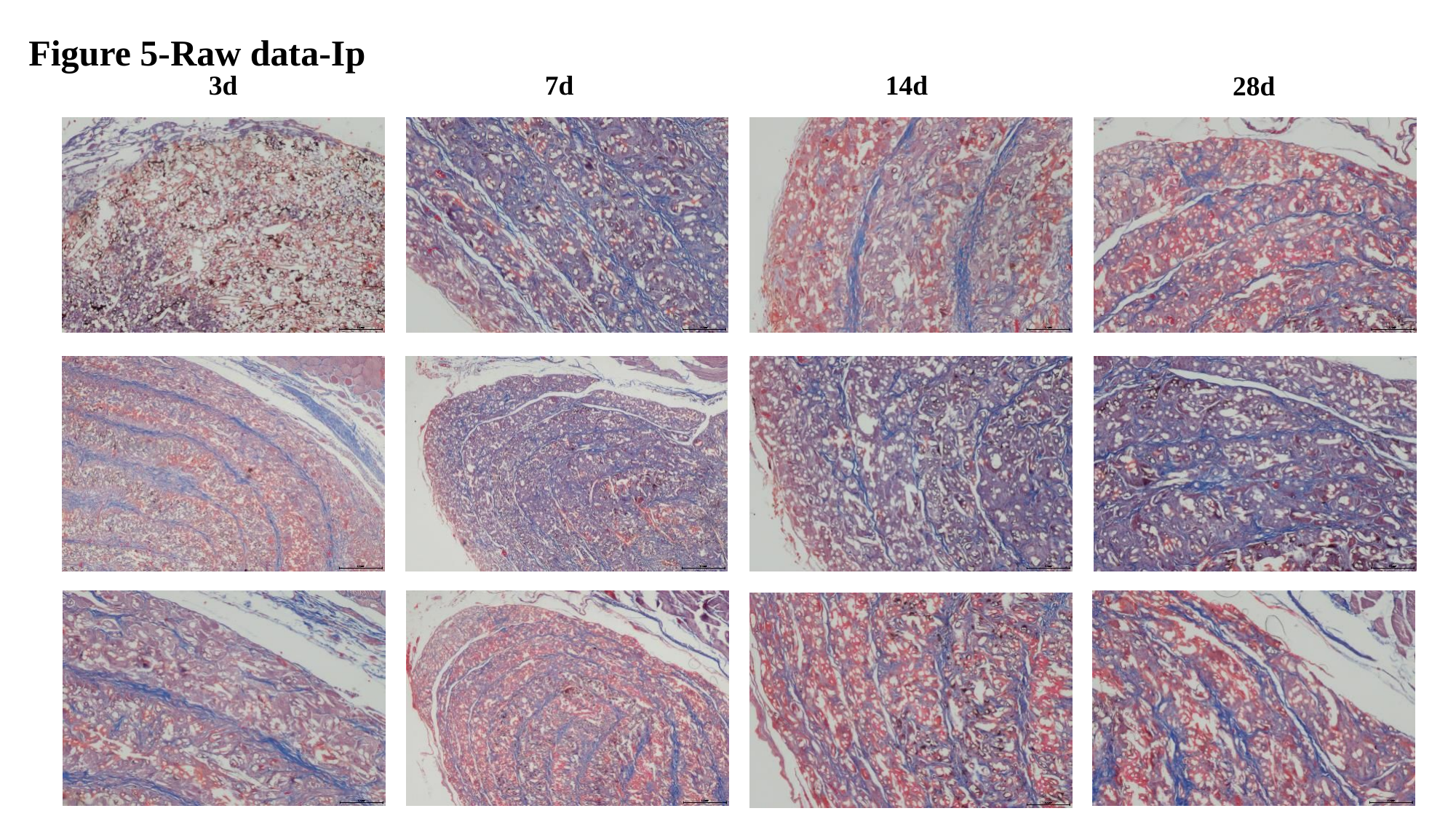

Figure 5-Raw data-Ip
3d
7d
14d
28d

## Slide 11
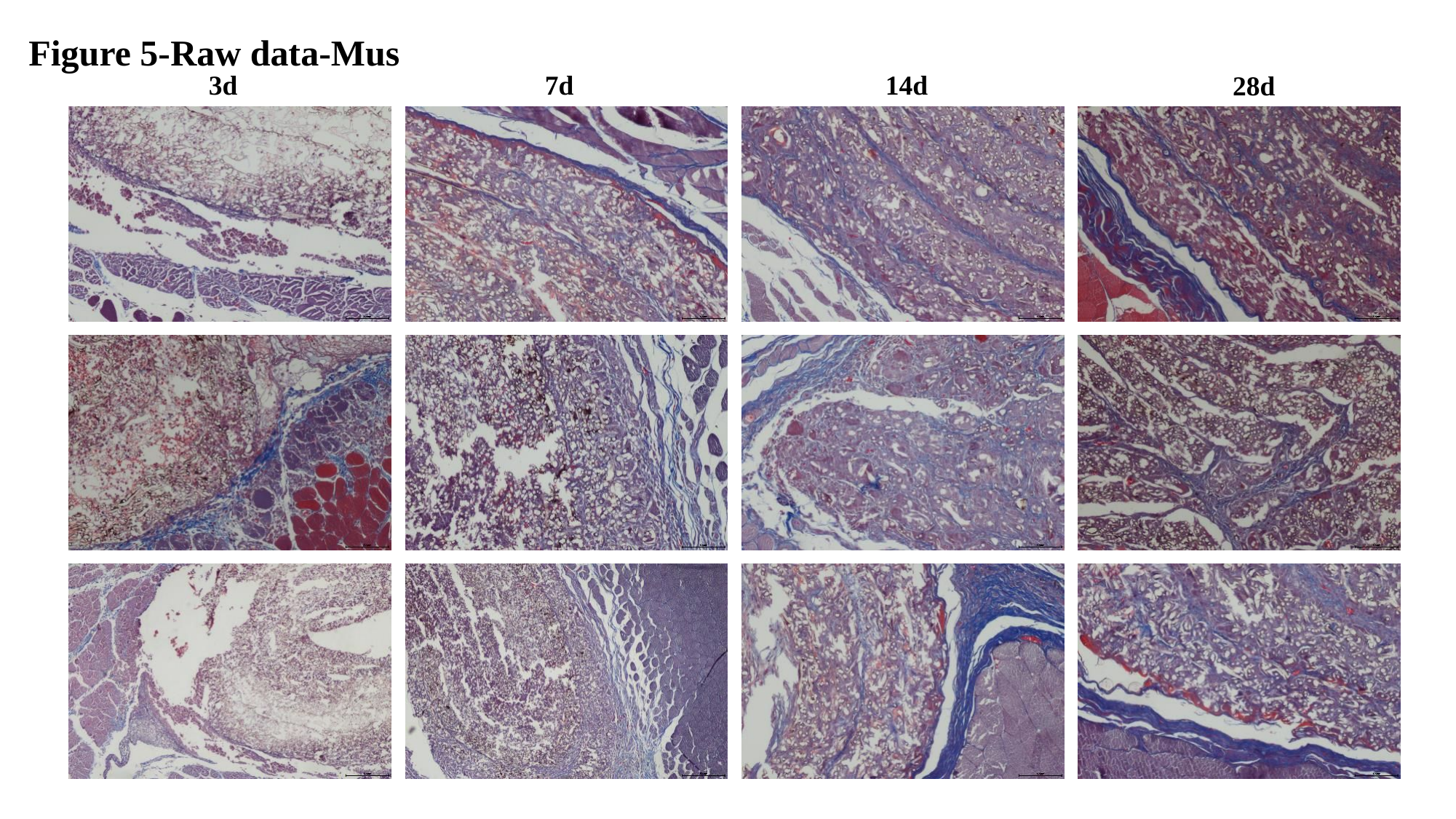

Figure 5-Raw data-Mus
3d
7d
14d
28d

## Slide 12
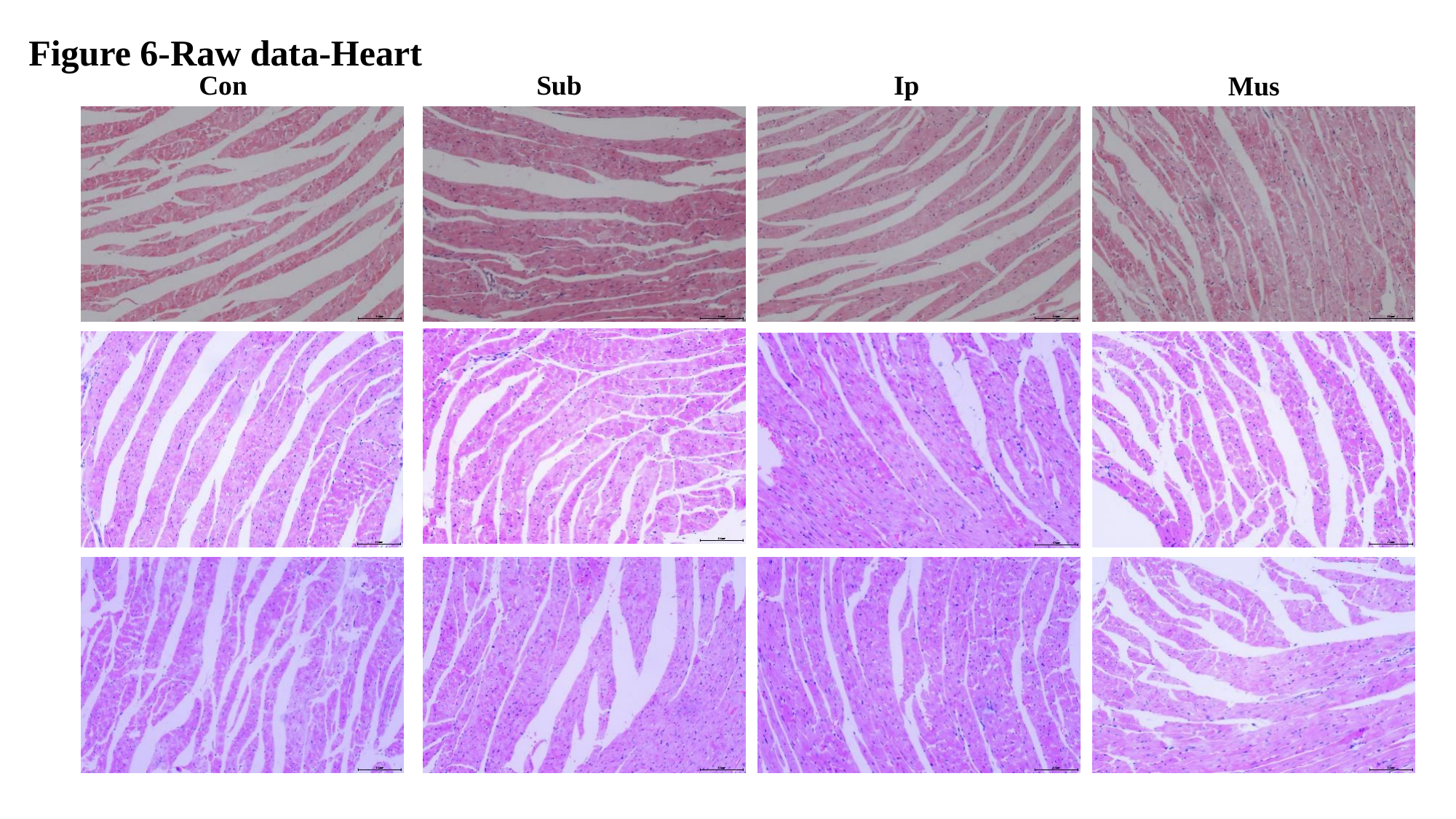

Figure 6-Raw data-Heart
Con
Sub
Ip
Mus

## Slide 13
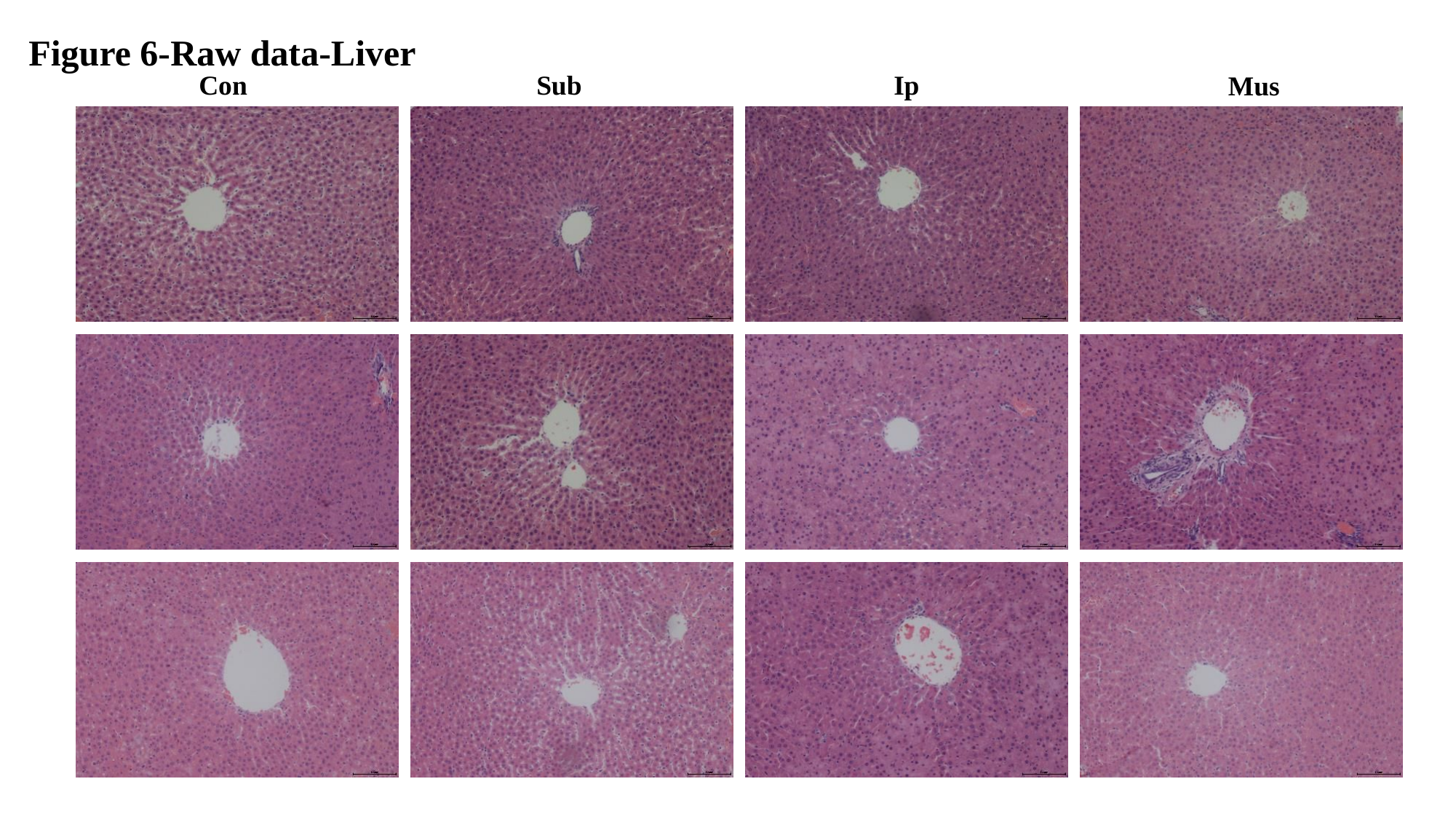

Figure 6-Raw data-Liver
Con
Sub
Ip
Mus

## Slide 14
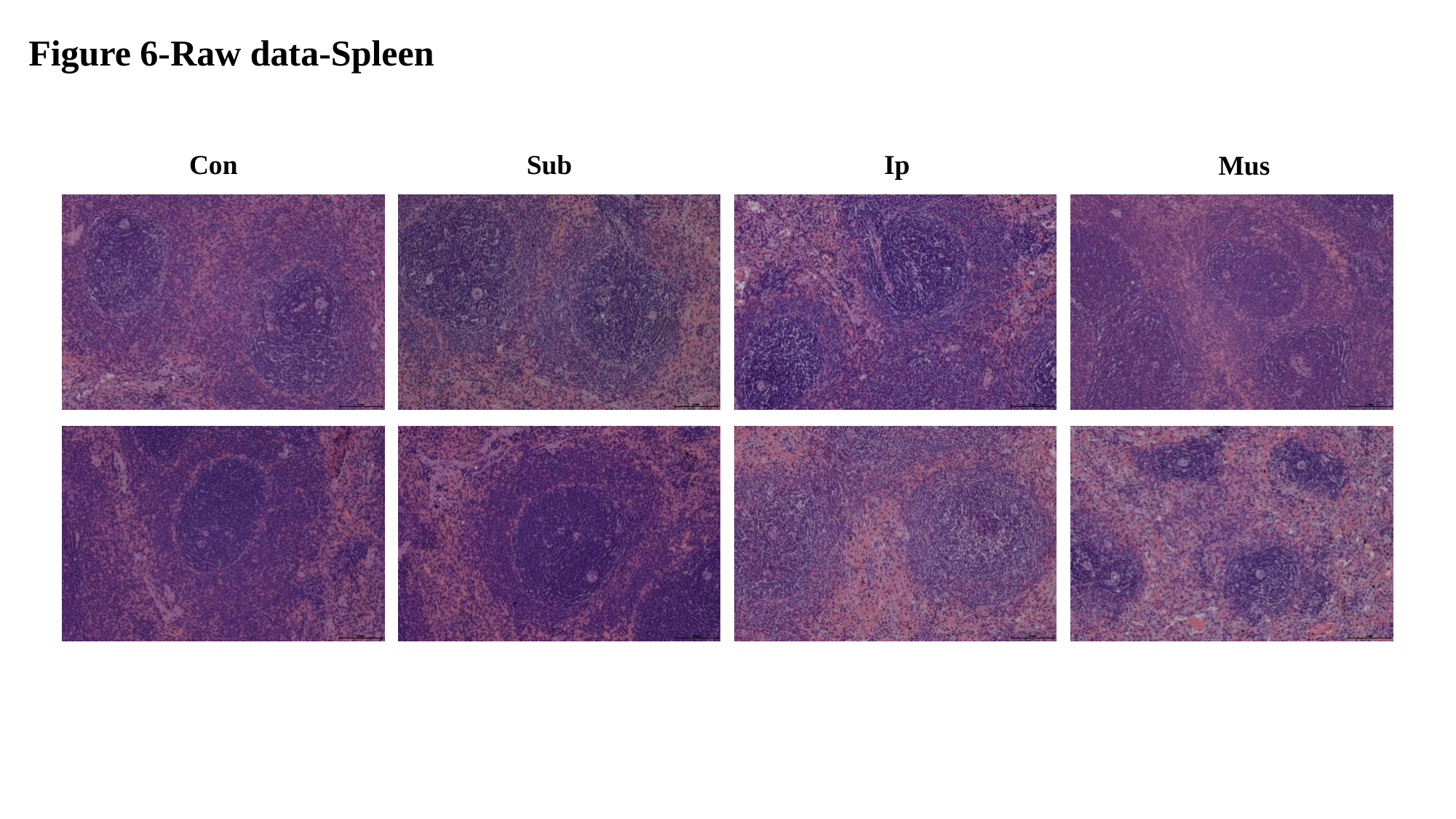

Figure 6-Raw data-Spleen
Con
Sub
Ip
Mus

## Slide 15
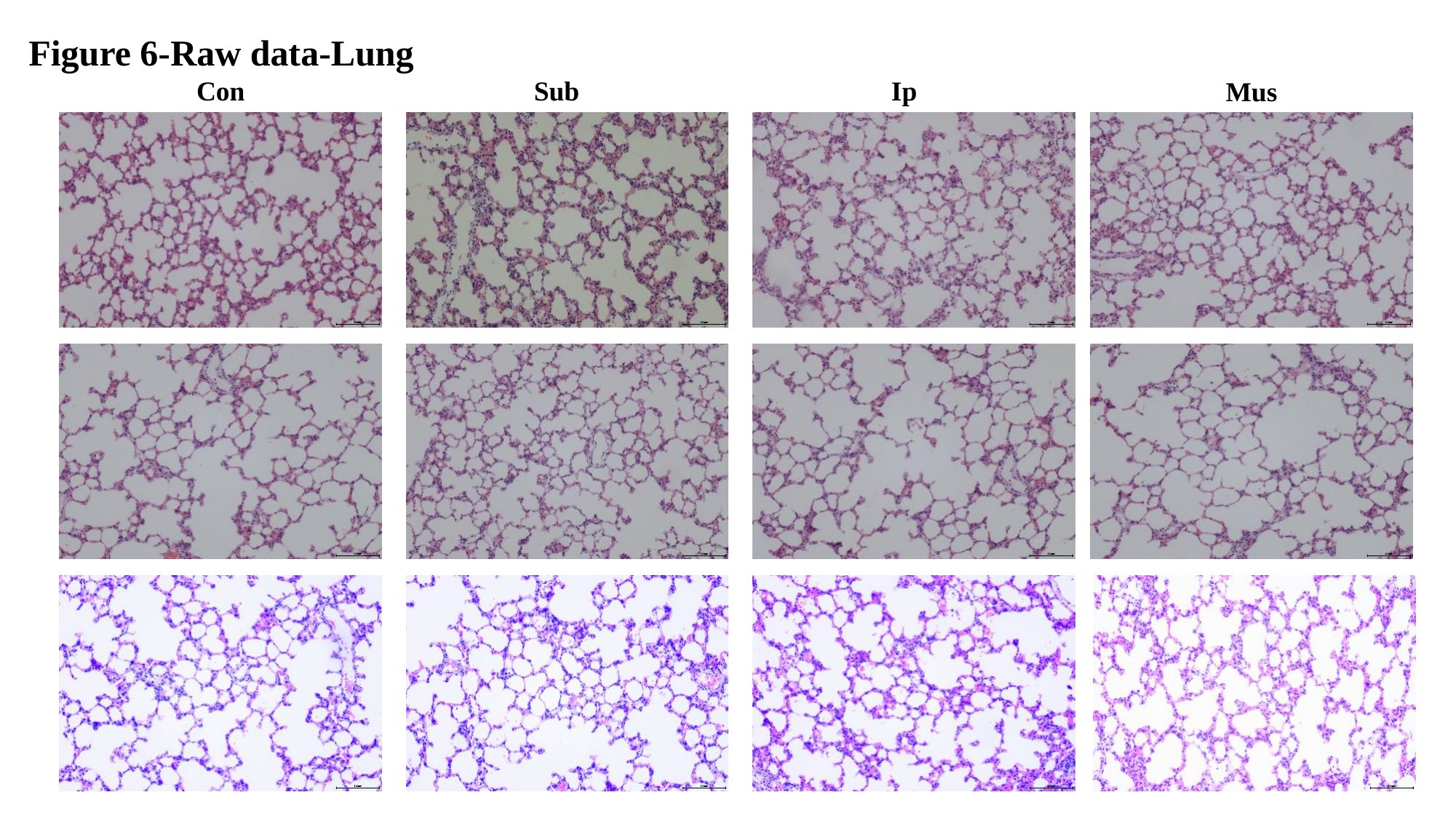

Figure 6-Raw data-Lung
Con
Sub
Ip
Mus

## Slide 16
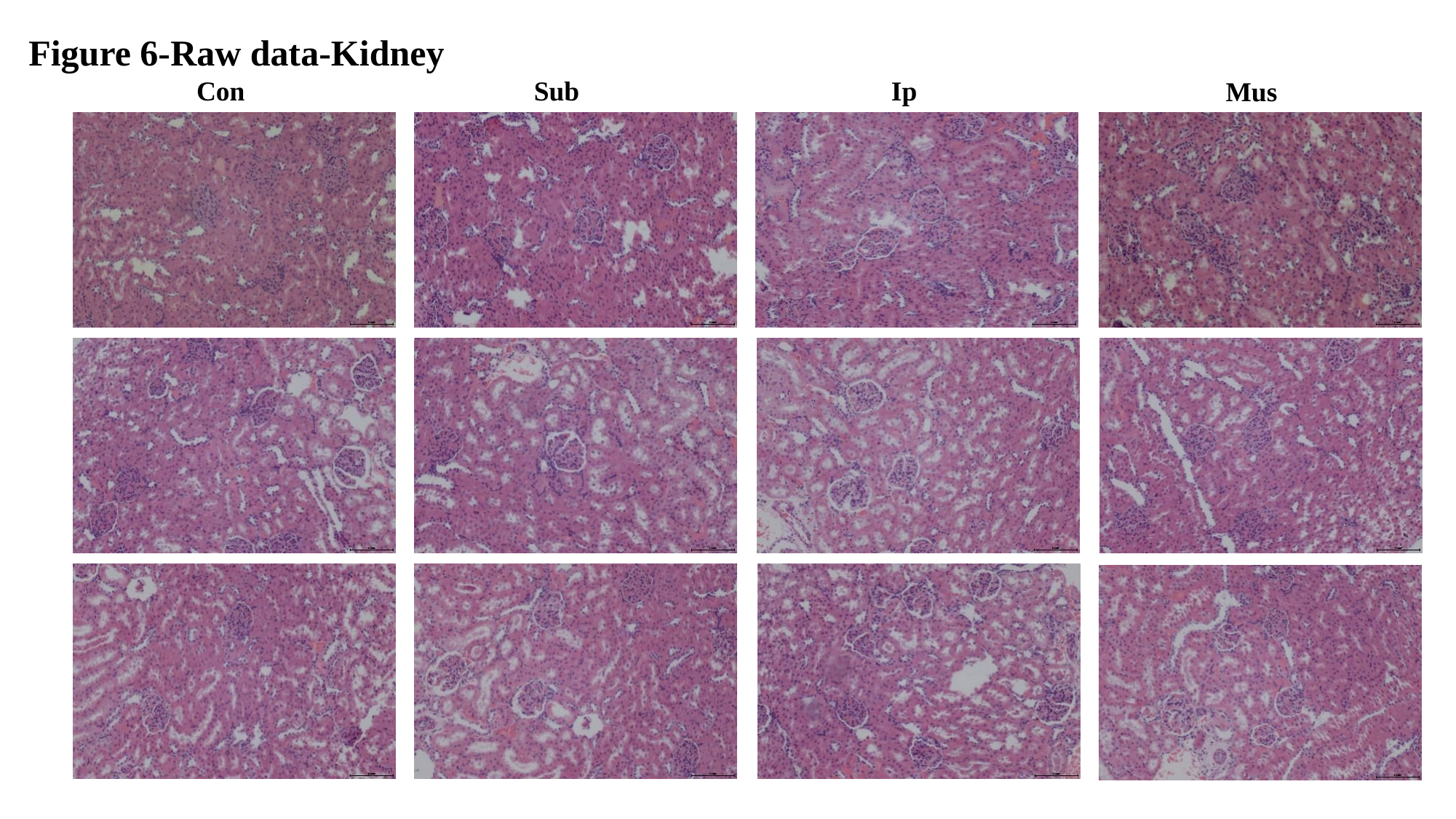

Figure 6-Raw data-Kidney
Con
Sub
Ip
Mus

## Slide 17
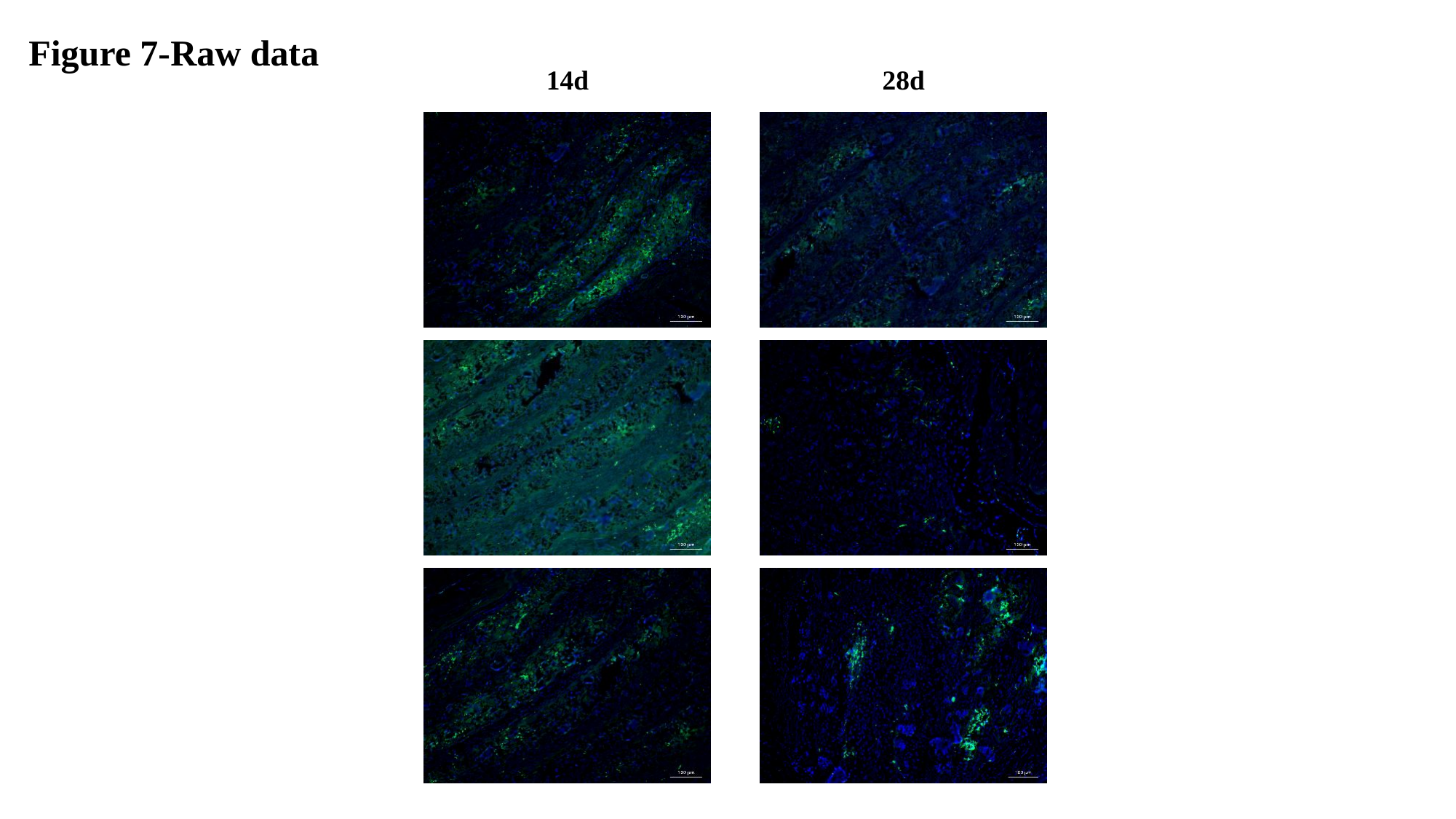

Figure 7-Raw data
14d
28d

## Slide 18
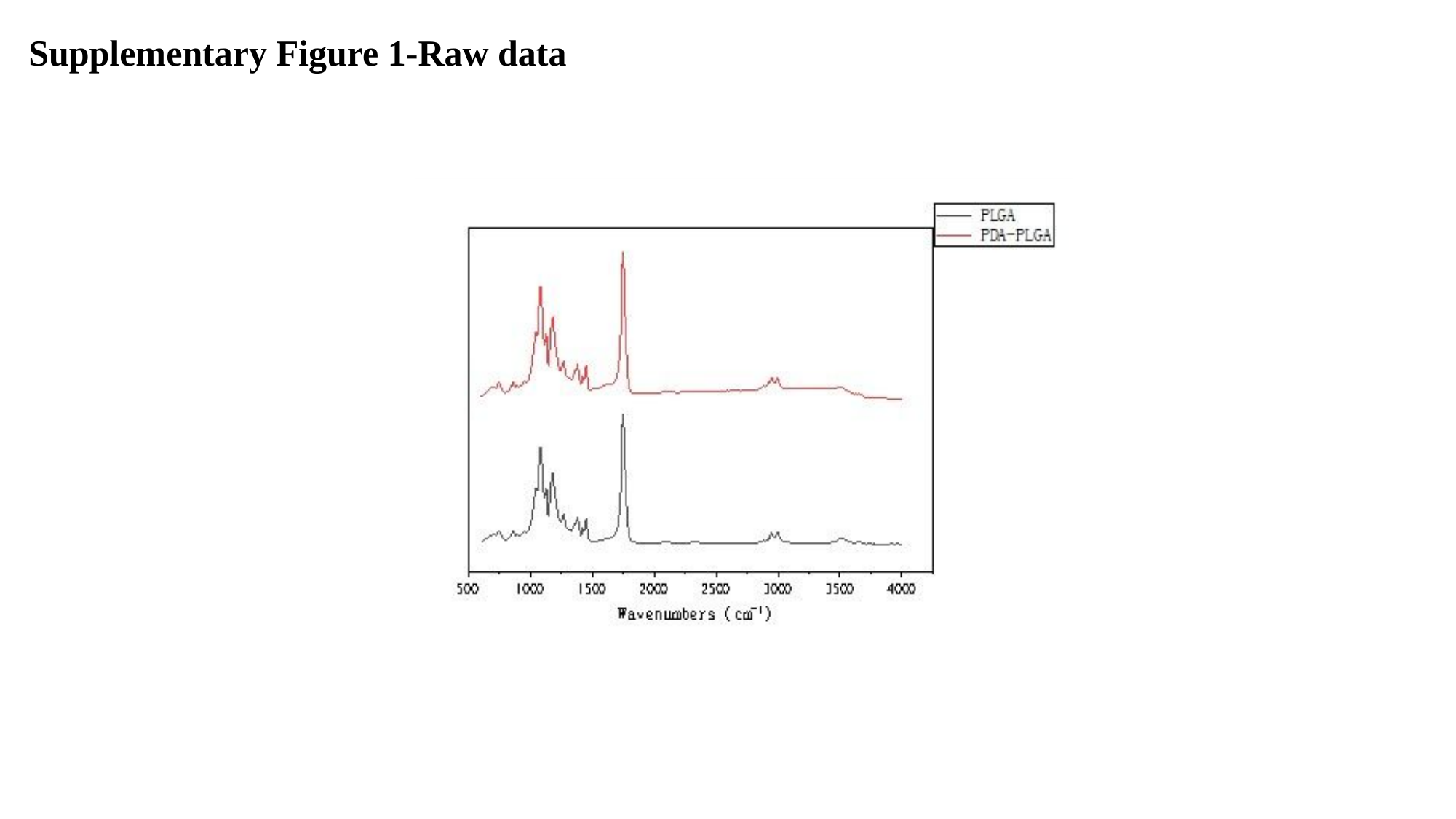

Supplementary Figure 1-Raw data

## Slide 19
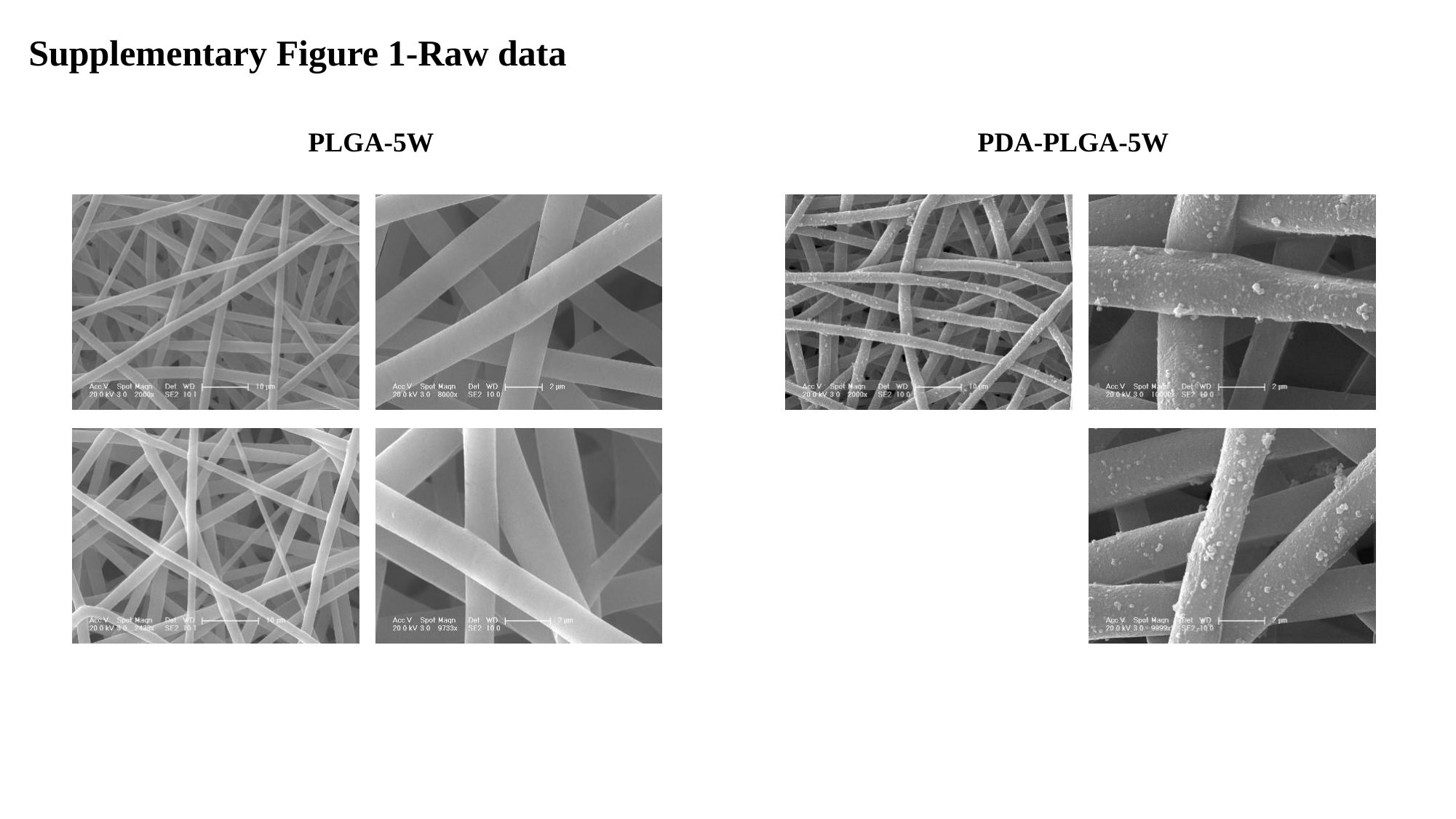

Supplementary Figure 1-Raw data
PDA-PLGA-5W
PLGA-5W

## Slide 20
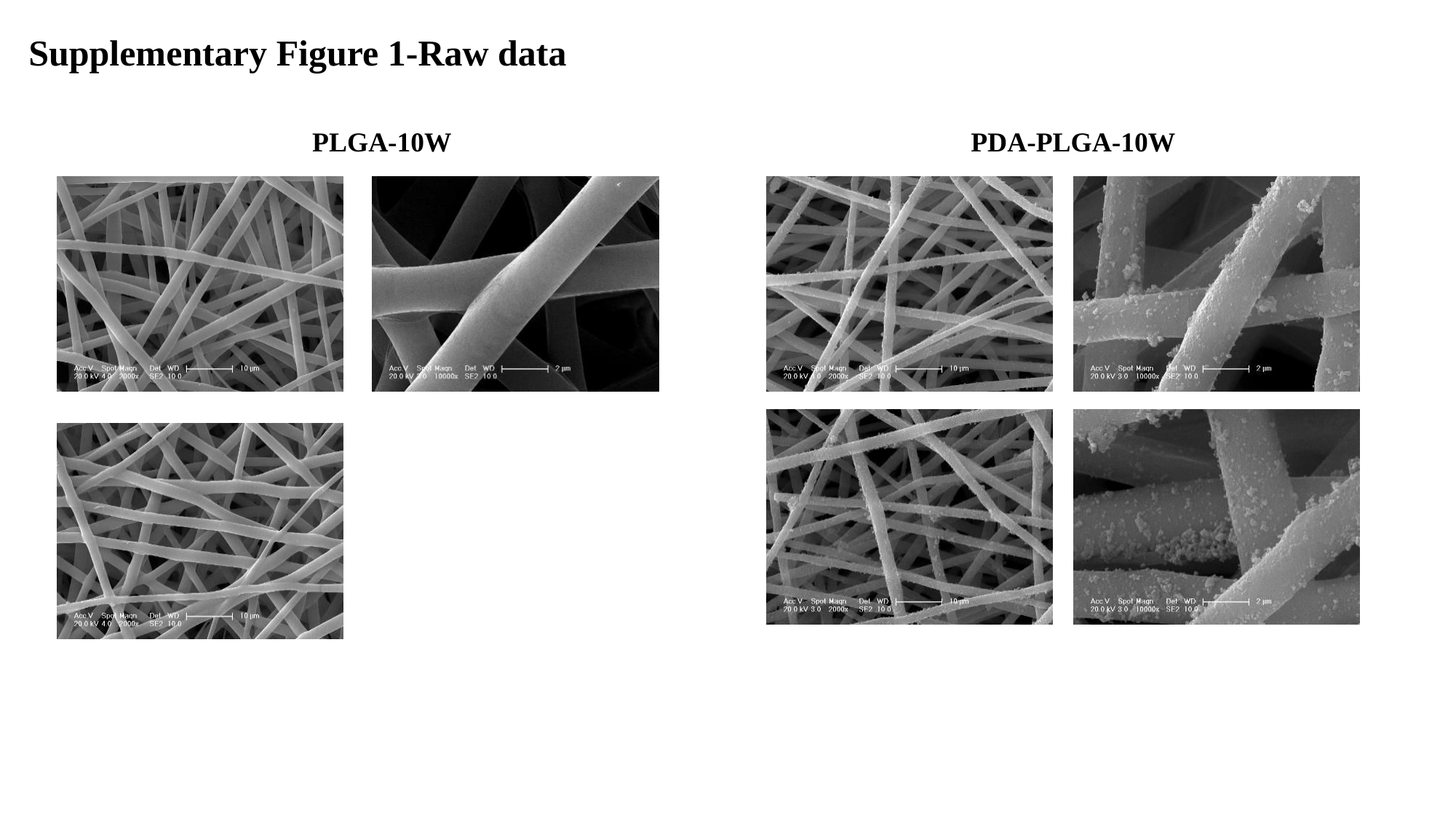

Supplementary Figure 1-Raw data
PDA-PLGA-10W
PLGA-10W
