# Supplementary material for: Study on the Effect of PDA-PLGA Scaffold Loaded With Islet Cells for Skeletal Muscle Transplantation in the Treatment of Diabetes
Source: Front Bioeng Biotechnol. 2022 Jun 30;10:927348. doi: 10.3389/fbioe.2022.927348 (PMC9280155; doi:10.3389/fbioe.2022.927348)

**Supplementary**

**Figure1.** The characterization of PDA-PLGA scaffolds.

A: Fourier Infrared Spectroscopy. B: Scanning electron microscopy (including partial magnification); a: PLGA electrospun membrane (Mw = 50 kDa), b: PDA-PLGA electrospun membrane (Mw = 50 kDa), c: PLGA electrospun membrane (Mw = 100 kDa), d: PDA-PLGA electrospun membrane (Mw = 100 kDa). C: Water contact angle, PLGA-5W: PLGA electrospun membrane (Mw = 50 kDa), PDA-PLGA-5W: PDA-PLGA electrospun membrane (Mw = 50 kDa), PLGA-10W: PLGA electrospun membrane (Mw = 100 kDa), PDA-PLGA-10W: PDA-PLGA electrospun membrane (Mw = 100 kDa). n=3, ^***^p<0.001 compared to PLGA-5W group; ^###^p<0.001 compared with PLGA-10W group. D and E: Tensile strength and elastic modulus. n=3, ^*^p<0.05 compared with PLGA-5W group; ^#^p<0.05 compared with PLGA-10W group; ^&&^p<0.01 compared with PDA-PLGA-5W group. F: Lactic acid content of degradation products, n=3, ^**^p<0.01 compared with PLGA-5W group.


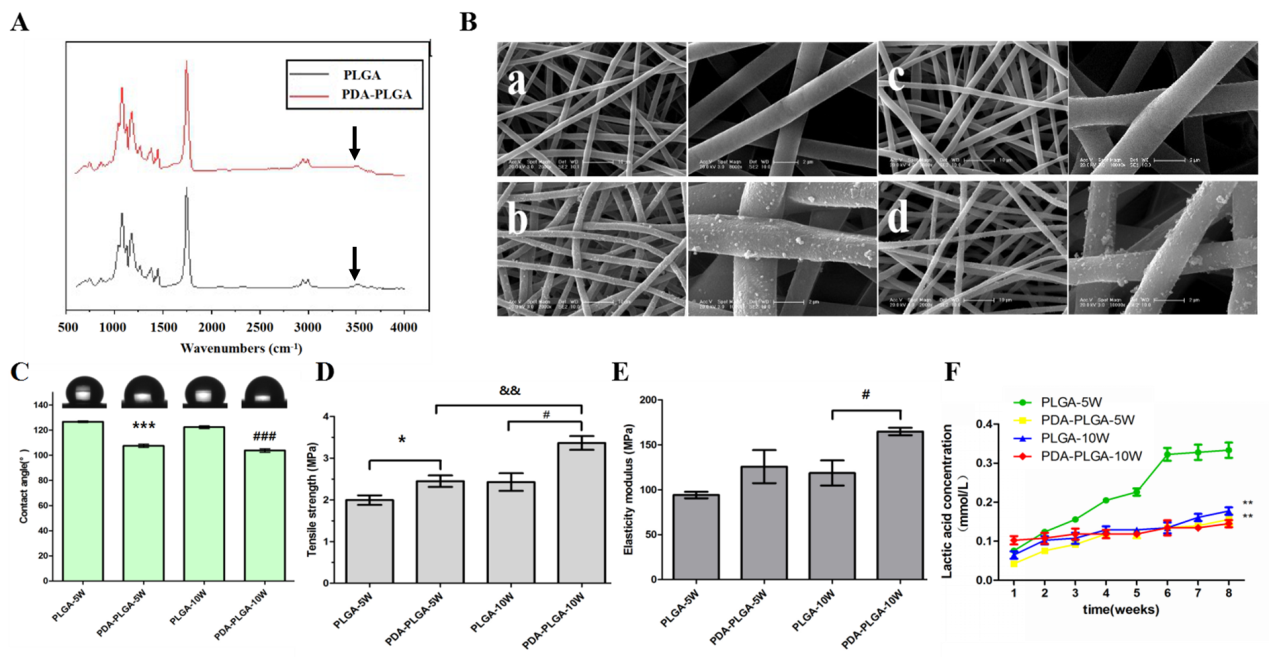

Supplement: Supplementary file 2 [file DataSheet1.DOCX]
